# Supplementary material for: PRMT6-mediated H3R2me2a guides Aurora B to chromosome arms for proper chromosome segregation
Source: Nat Commun. 2020 Jan 30;11:612. doi: 10.1038/s41467-020-14511-w (PMC6992762; doi:10.1038/s41467-020-14511-w)
Supplement: Supplementary file 1 — Supplementary Information [file 41467_2020_14511_MOESM1_ESM.pdf]

Supplementary Information

PRMT6-mediated H3R2me2a guides Aurora B to chromosome arms for proper chromosome segregation

Kim et al.

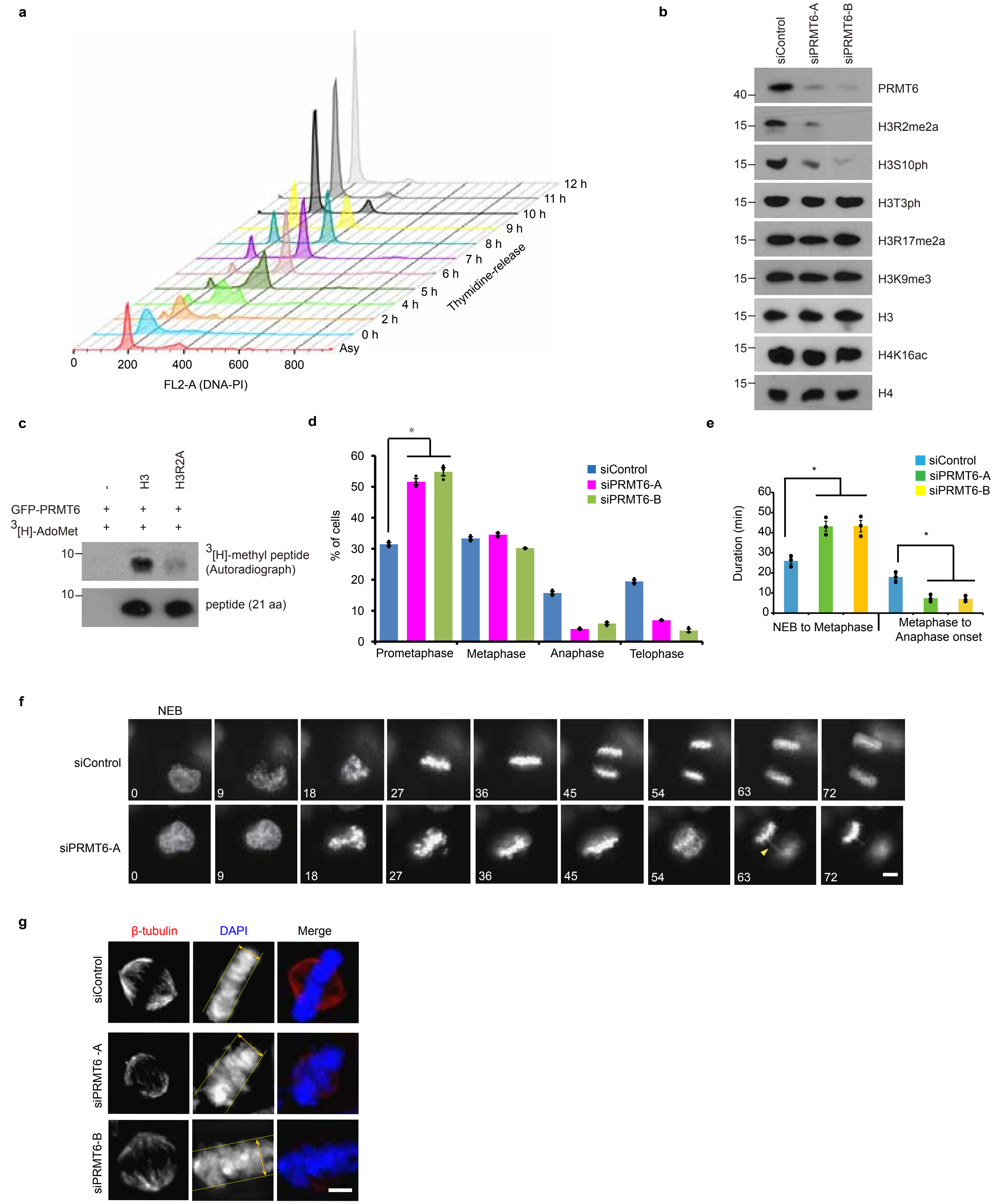

Supplementary Figure 1. Mitotic progression in PRMT6-depleted cells. (a) Thymidine-released cells were stained with propidium iodide (PI) and phospho-MPM2 antibody, and the cell cycle stages were analyzed by fluorescence-activated cell sorting (FACS). **(b)** Seventy-two hours after transfection with PRMT6-specific siRNA, MCF7 cells were harvested and the levels of the indicated proteins were analyzed with Western blotting. **(c)** *In vitro* PRMT6 methyltransferase assay. **(d)** The frequencies of the different mitotic phases were analyzed in PRMT6-depleted cells (n = 300 mitotic cells from three independent experiments). **(e,f)** Forty-eight hours after transfection, the HeLa cells stably expressing GFP-histone H2B were filmed for five hours. The duration of mitotic progression was determined (n = 150 cells from three independent experiments). **(g)** The images are maximum projections from z stacks of PRMT6-depleted cells stained for β-tubulin (red) and DNA (blue). The bidirectional arrows represent the width of the metaphase plate. Scale bars, 5 μm. Error bars, SEMs. Source data are provided as a Source Data file. (Student's t-test $*p < 0.01$).

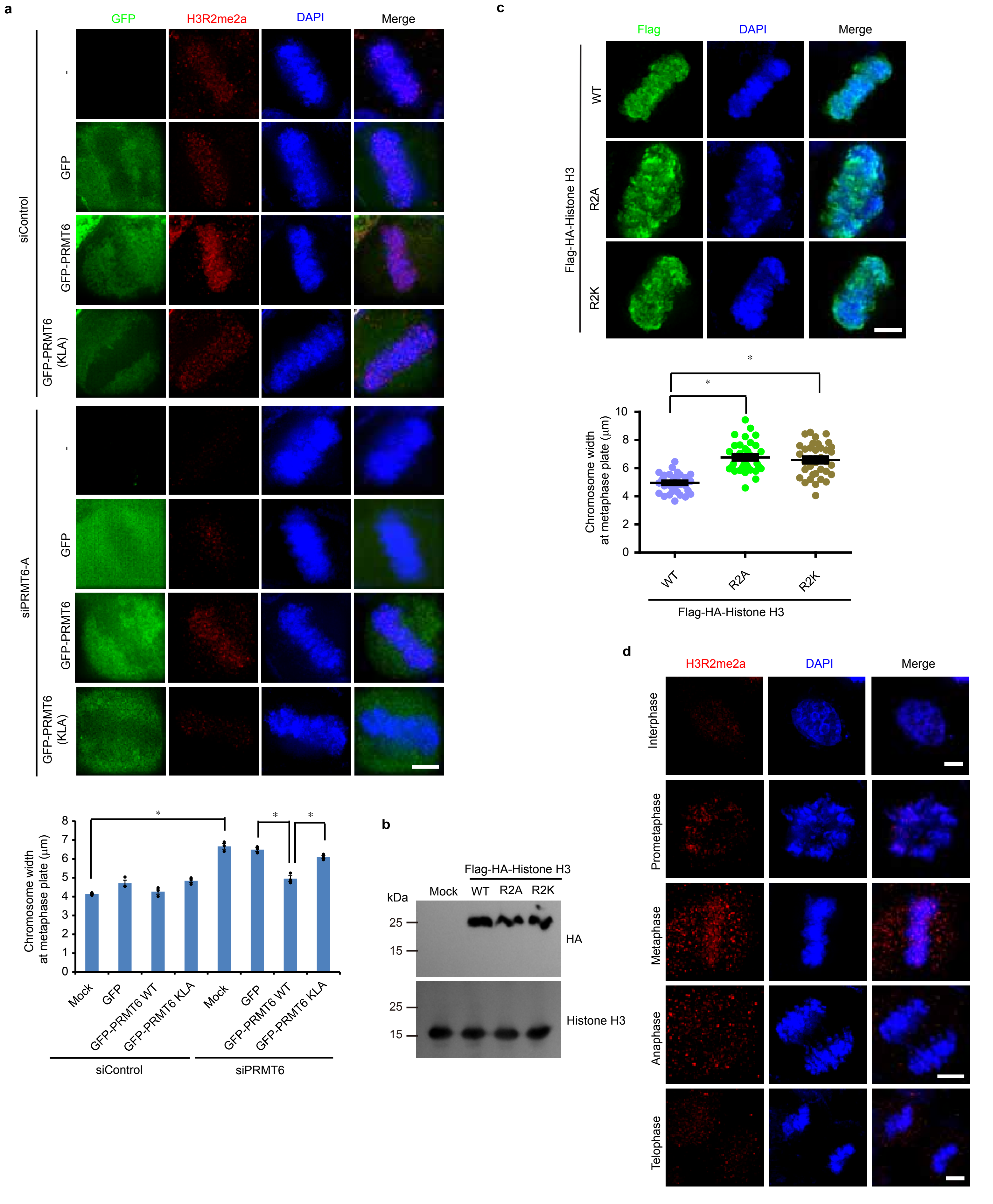

Supplementary Figure 2. H3R2 methylation by PRMT6 is necessary for chromosome integrity. (a) Forty-eight hours after siRNA transfection, the HeLa cells were transfected with the indicated plasmids and incubated for 28 hours prior to fixation with MeOH and staining with antibodies against H3R2me2a. The width of metaphase plate was measured and plotted (n = 30 metaphase cells from three independent experiments). **(b, c)** Twenty-eight hours after Flag-Histone H3 WT or mutant (R2A or R2K) transfection, the HeLa cells were fixed with MeOH and harvested for Western blotting or staining with the indicated antibodies. The width of the metaphase plate in Flag-positive cells was measured and plotted (n = 36 metaphase cells from three independent experiments). **(d)** Maximum projections from deconvolved z stacks of representative HeLa cells stained for H3R2me2a (red) and DNA (blue) are shown. Scale bars, 5 µm. Error bars, SEMs. P values were calculated by two-way ANOVA (a; *$p < 0.01$) or two-tailed Student's t-test (c; *$p < 0.01$). Source Data are provided as a Source Data file.

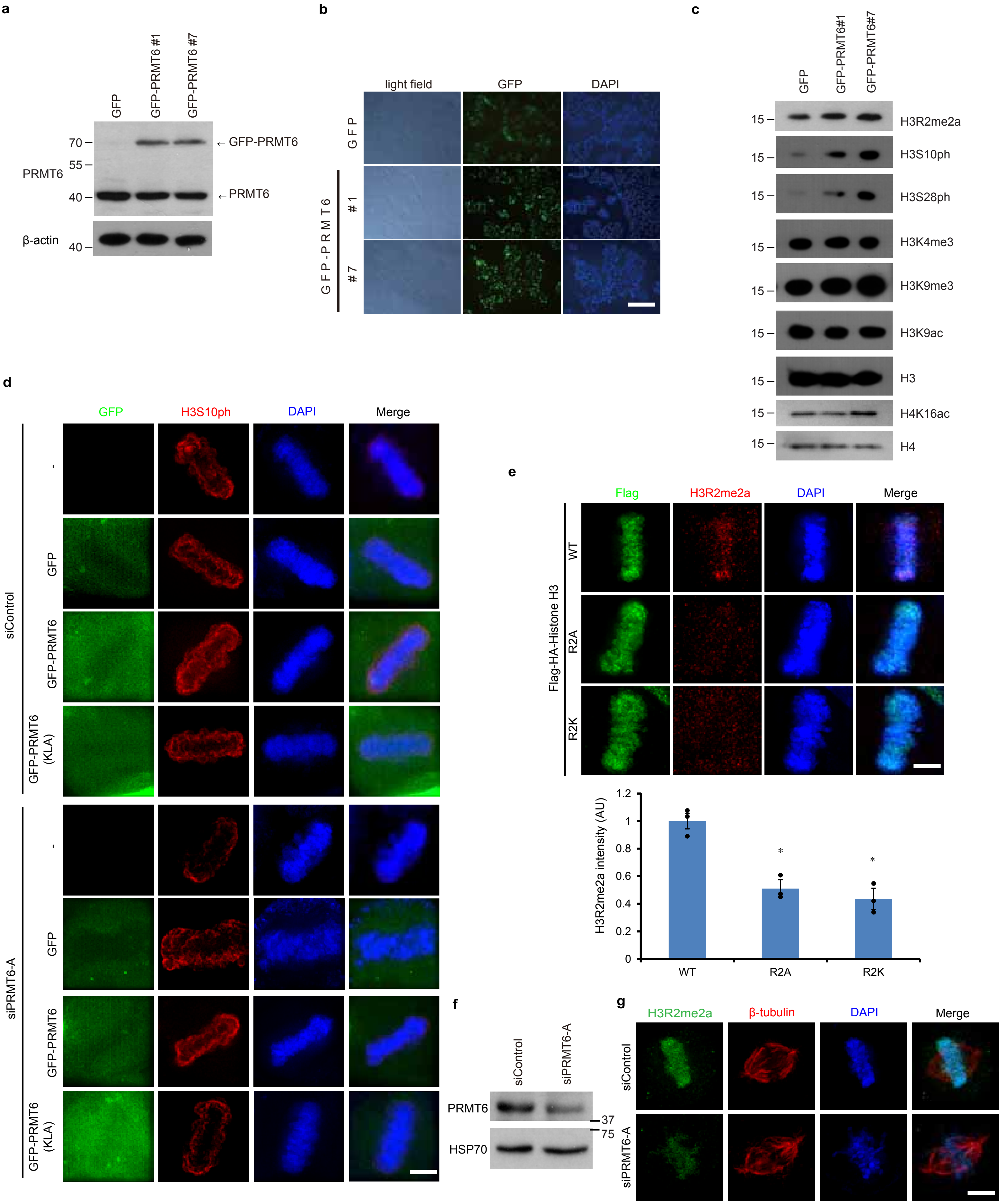

Supplementary Figure 3. H3R2 methylation by PRMT6 is necessary for H3S10 phosphorylation. (a-c) Two clones of MCF7 cells stably expressing GFP-PRMT6 were harvested and subjected to Western blotting with the indicated antibodies. Actin served as the loading control. The expression of GFP or GFP-PRMT6 was confirmed with fluorescence microscopy (b). Scale bar, 100 µm. **(d)** Forty-eight hours after siRNA transfection, the HeLa cells were transfected with the indicated plasmids and incubated for 28 hours prior to fixation with MeOH and staining with antibodies against H3S10ph. **(e)** Twenty-eight hours after Flag-Histone H3 WT or mutant (R2A or R2K) transfection, HeLa cells were fixed with MeOH and stained with the indicated antibodies. The intensity of H3R3me2a in Flag-positive cells was measured and plotted (n = 30 metaphase cells from three independent experiments). **(f, g)** Seventy two hours after transfection with siPRMT6, RPE1 cells were harvested to analyze the levels of the indicated proteins with Western blotting (f) or were stained with the indicated antibodies (g). Scale bars, 5 µm. Error bars, SEM. Source data are provided as a Source Data file. (Student's t-test *p < 0.01).

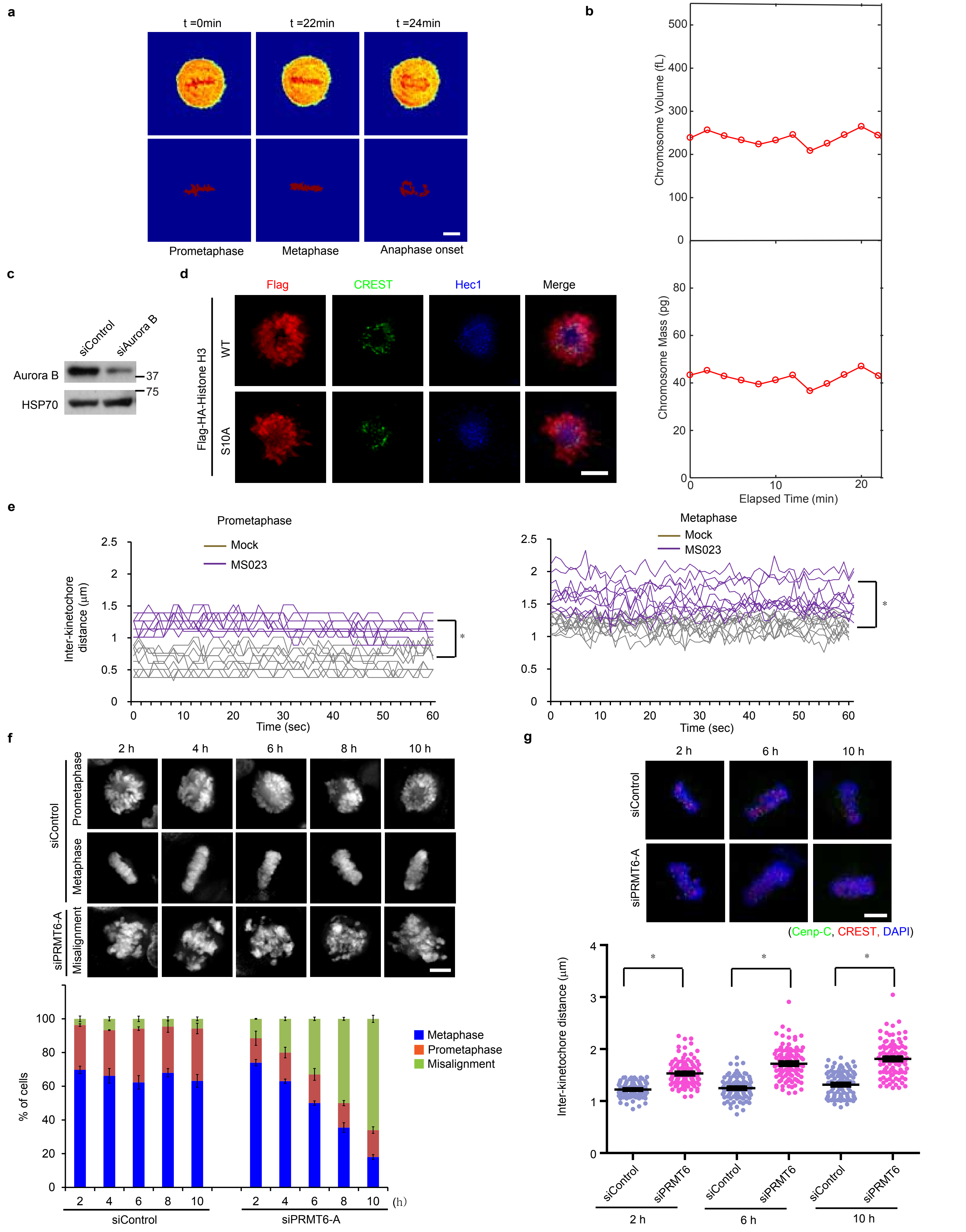

Supplementary Figure 4. Analysis of chromosome integrity. (a, b) The HeLa cells were subjected to time-lapse imaging with tomographic microscopy at every 2-min intervals to calibrate of the volume and mass of the mitotic chromosomes. The volume and mass are plotted in (b). **(c)** Forty-eight hours after siAurora B transfection, HeLa cells were harvested, and the levels of the indicated proteins were analyzed. **(d)** Twenty-eight hours after Flag-histone H3 WT or S10A mutant transfection, cells were stained with the indicated antibodies. **(e)** HeLa/GFP-CenpA cells were treated with a PRMT6 inhibitor and subjected to time-lapse imaging with confocal microscopy for GFP-CenpA starting at 24 hours after inhibitor treatment. The distance between paired kinetochores was determined for control and PRMT6-depleted cells from three independent

experiments (n=10 kinetochore pairs). **(f)** HeLa cells were exposed to 0.5 μM MG132 for the indicated duration and fixed with MeOH for DNA staining, and the prometaphase and metaphase cells in 200 mitotic cells from four independent experiments was quantified. **(g)** Seventy two hours after transfection with siPRMT6, the HeLa cells were exposed to 0.5 μM MG132, fixed at the indicated time points, and stained for Cenp-C (green), CREST (red), and DNA (blue). The inter-KT distance was measured in more than 100 kinetochore pairs in metaphase cells from three independent experiments. Scale bars, 5 μm. Error bars, SEMs. Source data are provided as a Source Data file. (Student's t-test $*p < 0.01$).

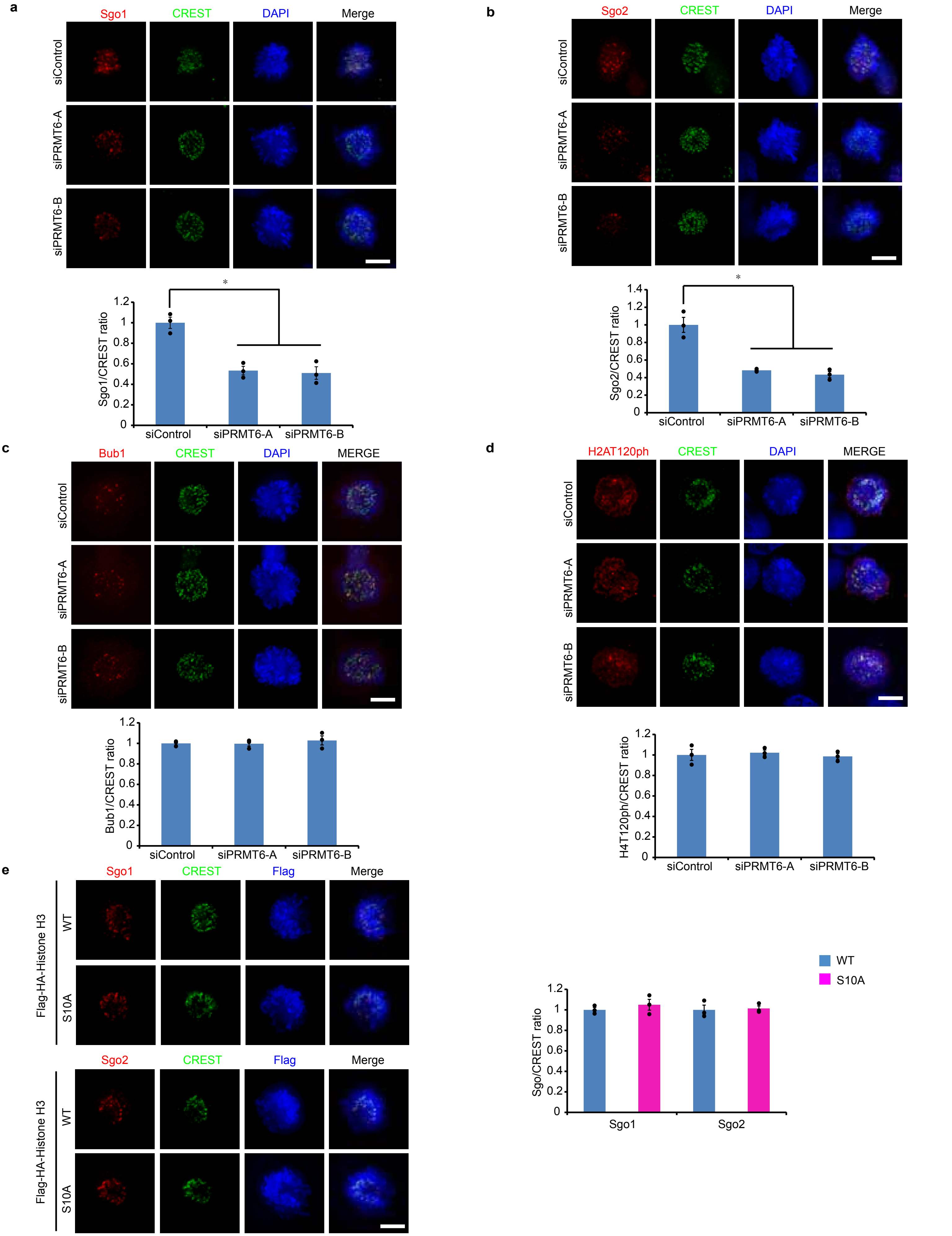

Supplementary Figure 5. Analysis of centromere integrity. (a-d) Seventy-two hours after transfection with siPRMT6, the HeLa cells were stained with the indicated antibodies. The fluorescence intensity ratios of centromeric Sgo1/CREST (a), Sgo2/CREST (b), Bub1/CREST (c), and H2AT120ph/CREST (d) in prometaphase cells were quantified and plotted (n = 300 centromeres from three independent experiments). **(e)** Twenty-eight hours after Flag-histone H3 WT or S10A mutant transfection, cells were stained with the indicated antibodies, and the fluorescence intensity ratios of Sgo1/CREST and Sgo2/CREST in Flag-positive prophase cells were quantified (n = 300 centromeres from three independent experiments). Scale bars, 5 µm. Error bars, SEMs. Source data are provided as a Source Data file. (Student's t-test *$p < 0.01$).

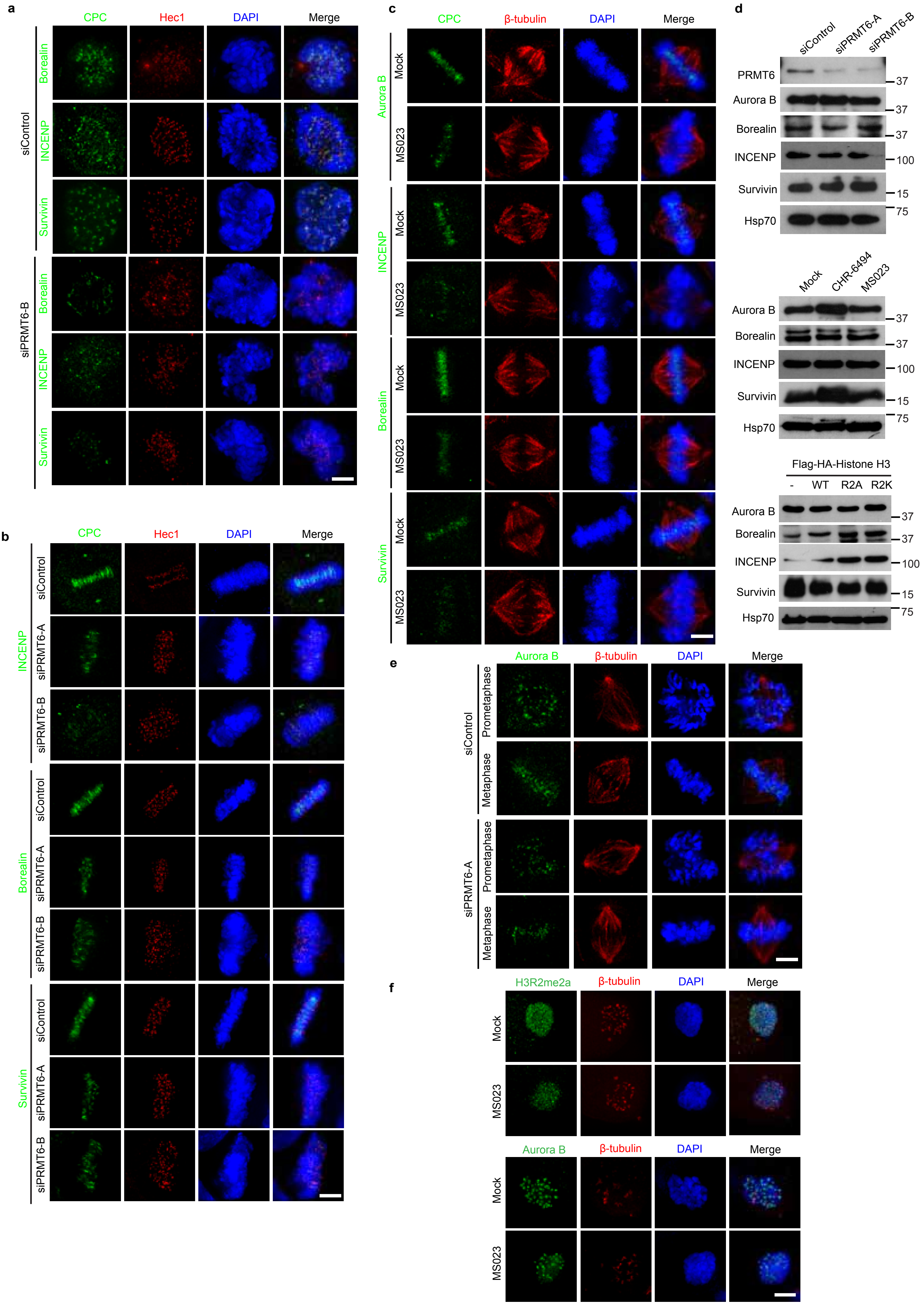

a
CPC
Hec1
DAPI
Merge
siControl
Borealin
INCENP
Survivin
siPRMT6-B
b
siPRMT6-A
c
β-tubulin
Aurora B
Mock
MS023
d
PRMT6
Hsp70
37
100
15
75
CHR-6494
Flag-HA-Histone H3
-
WT
R2A
R2K
e
Prometaphase
Metaphase
f
H3R2me2a

Supplementary Figure 6. Inhibition of H3R2 methylation decreased the targeting of CPC to centromeres. (a, b) The HeLa cells were treated with siPRMT6 for 72 hours, fixed with MeOH, and stained with the indicated antibodies. **(c)** The HeLa cells were treated with the PRMT6 inhibitor and stained with antibodies against CPC components including Aurora B, INCENP, Borealin, and Survivin in the centromeres. **(d)** The HeLa cells were transfected with PRMT6-specific siRNAs, Flag-HA-Histone H3 WT, or a mutant construct or were treated with the Haspin inhibitor or PRMT6 inhibitor. The levels of the indicated proteins were analyzed. (**e**) PRMT6-depleted RPE1 cells were stained with the indicated antibodies. **(f)** Eighteen hours after nocodazole-arrest, the HeLa cells were treated with the PRMT6 inhibitor for one hour and stained with the indicated antibodies. Scale bars, 5 µm. Source data are provided as a Source Data file.

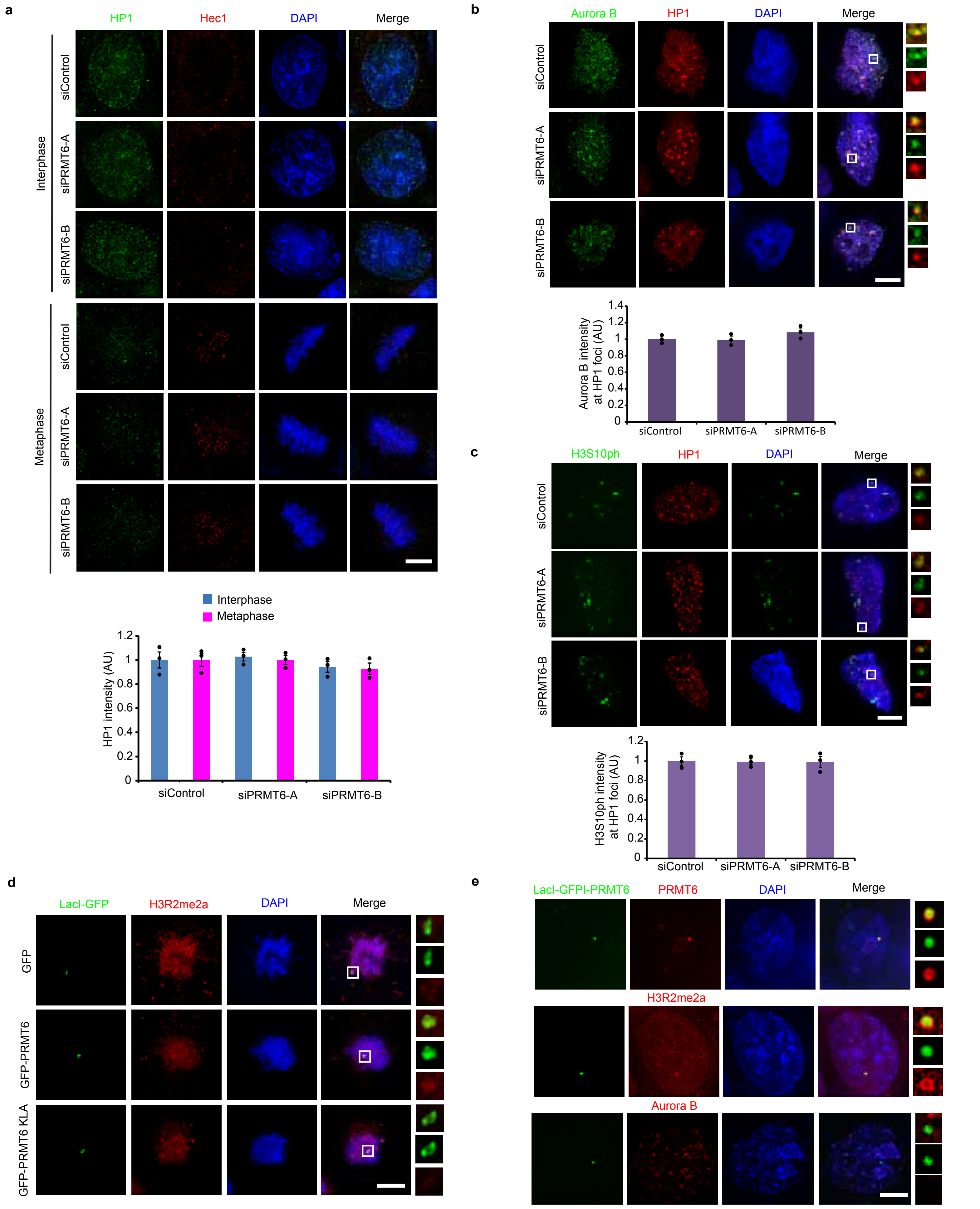

Supplementary Figure 7. H3R2 methylation recruits CPC to chromosomes in early mitosis. (a) Seventy-two hours after transfection with siPRMT6, HeLa cells were stained with the indicated antibodies. The intensity of the HP1 in interphase cells and metaphase cells was quantified and plotted (n = 30 cells from three independent experiments). **(b, c)** Forty eight hours after transfection with siPRMT6, the HeLa cells were treated with 1 µM RO3306 as a Cdk1 inhibitor for twenty-one hours and were stained with the indicated antibodies. The average intensities of the Aurora B and H3S10ph at HP1 foci in G2 cells were quantified and plotted (n = 150 foci from three independent experiments). **(d, e)** Twenty-eight hours after transfection with LacI-GFP-PRMT6, the LacO/TRE U2OS cells were fixed and stained with the antibodies against H3R2me2a, PRMT6, or Aurora B. The insets show single focal planes of the boxed regions. Scale bars, 5 µm. Error bars, SEMs. Source data are provided as a Source Data file.

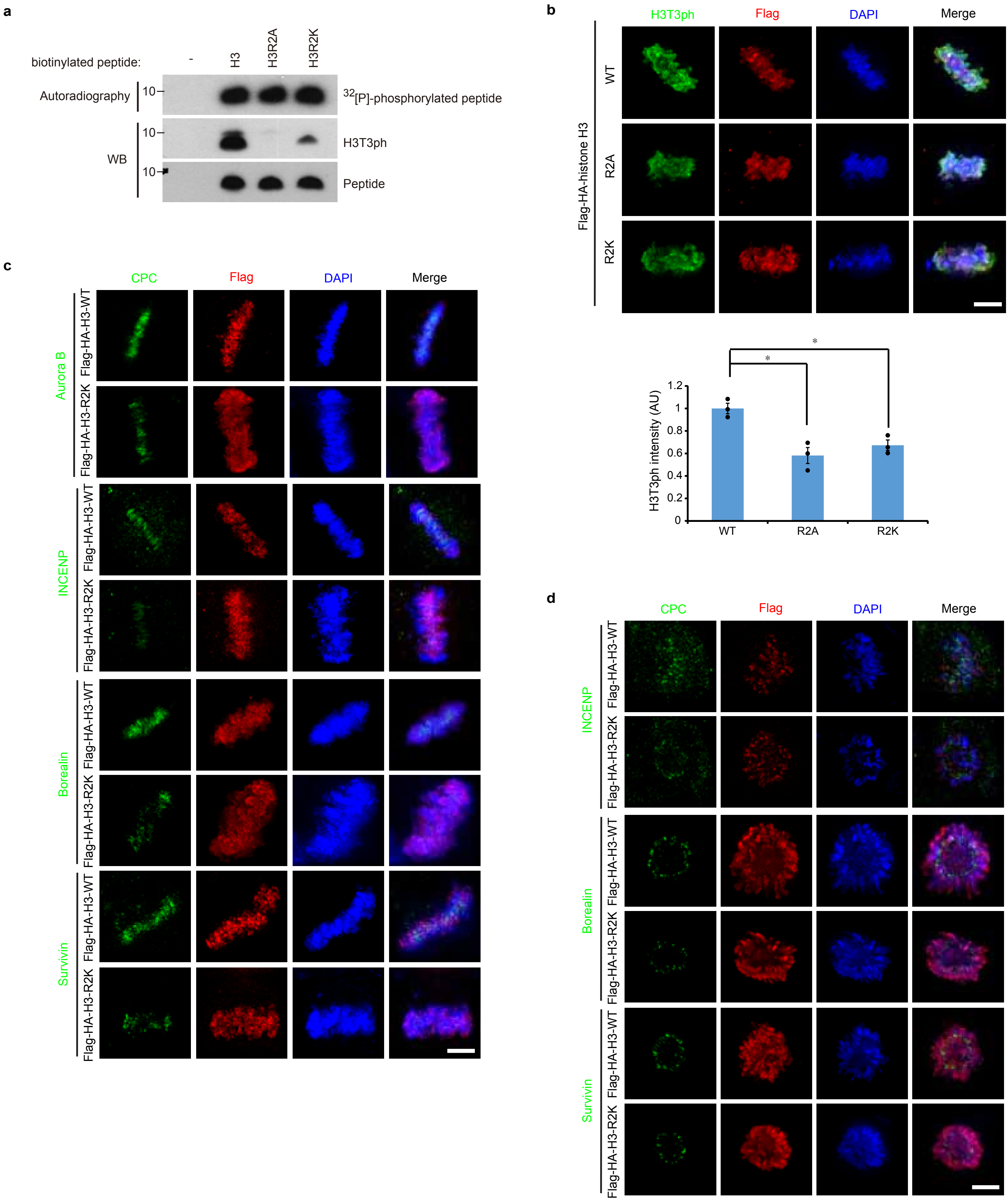

Supplementary Figure 8. H3R2me2a is a docking site for CPC on the chromosome arms. (a) After the *in vitro* Haspin kinase assay, the indicated peptides were subjected to autoradiography or Western blotting with the indicated antibodies. **(b-d)** Twenty-eight hours after Flag-Histone H3 WT or mutant (R2A or R2K) transfection, HeLa cells were fixed with MeOH and stained with the indicated antibodies. The images are maximum projections from z stacks of representative cell images, and they were acquired under a constant exposure time for all channels. The intensity of H3T3ph was analyzed and plotted (b, n = 30 metaphase cells from three independent experiments). Scale bars, 5 µm. Error bars, SEMs. Source data are provided as a Source Data file. (Student's t-test *$p < 0.01$).

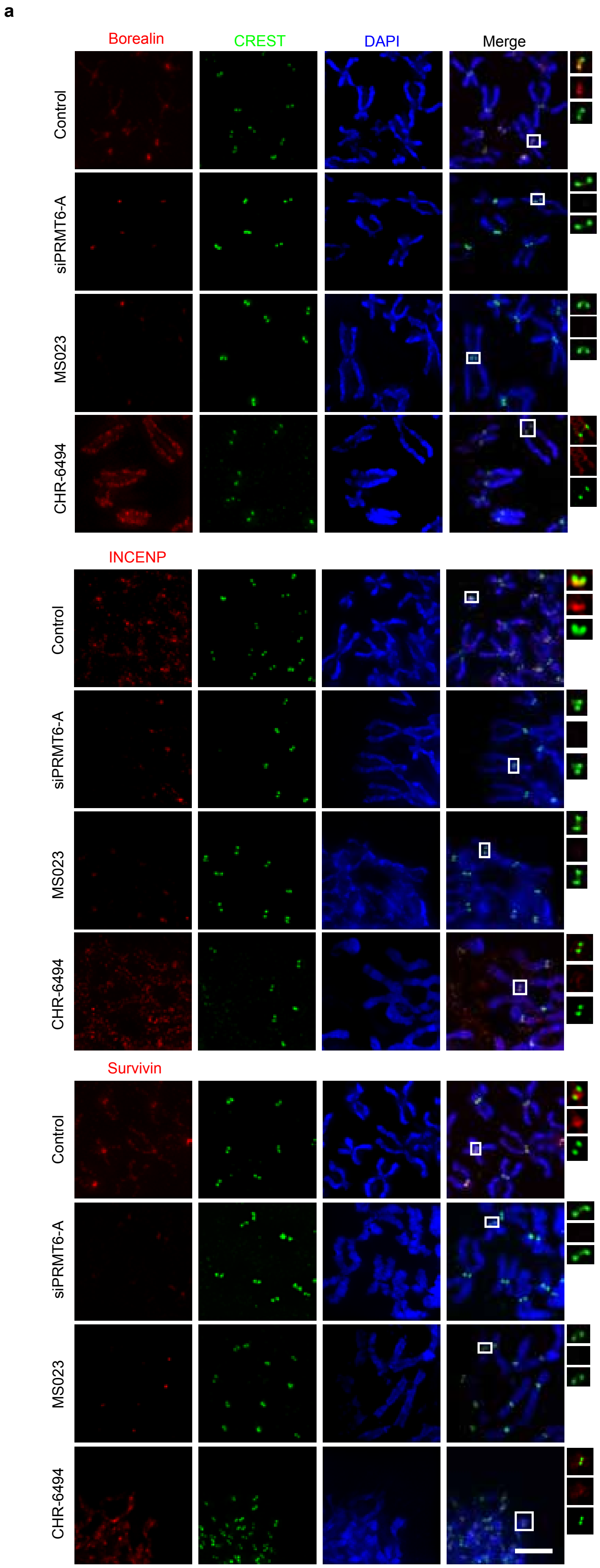

Supplementary Figure 9. H3R2me2a recruits CPC to the chromosome arm. (a) HeLa cells were treated with siPRMT6, the PRMT6 inhibitor, or the Haspin kinase inhibitor. The chromosome spreads from nocodazole-arrested HeLa cells were stained with the indicated antibodies. Scale bar, 5 µm.

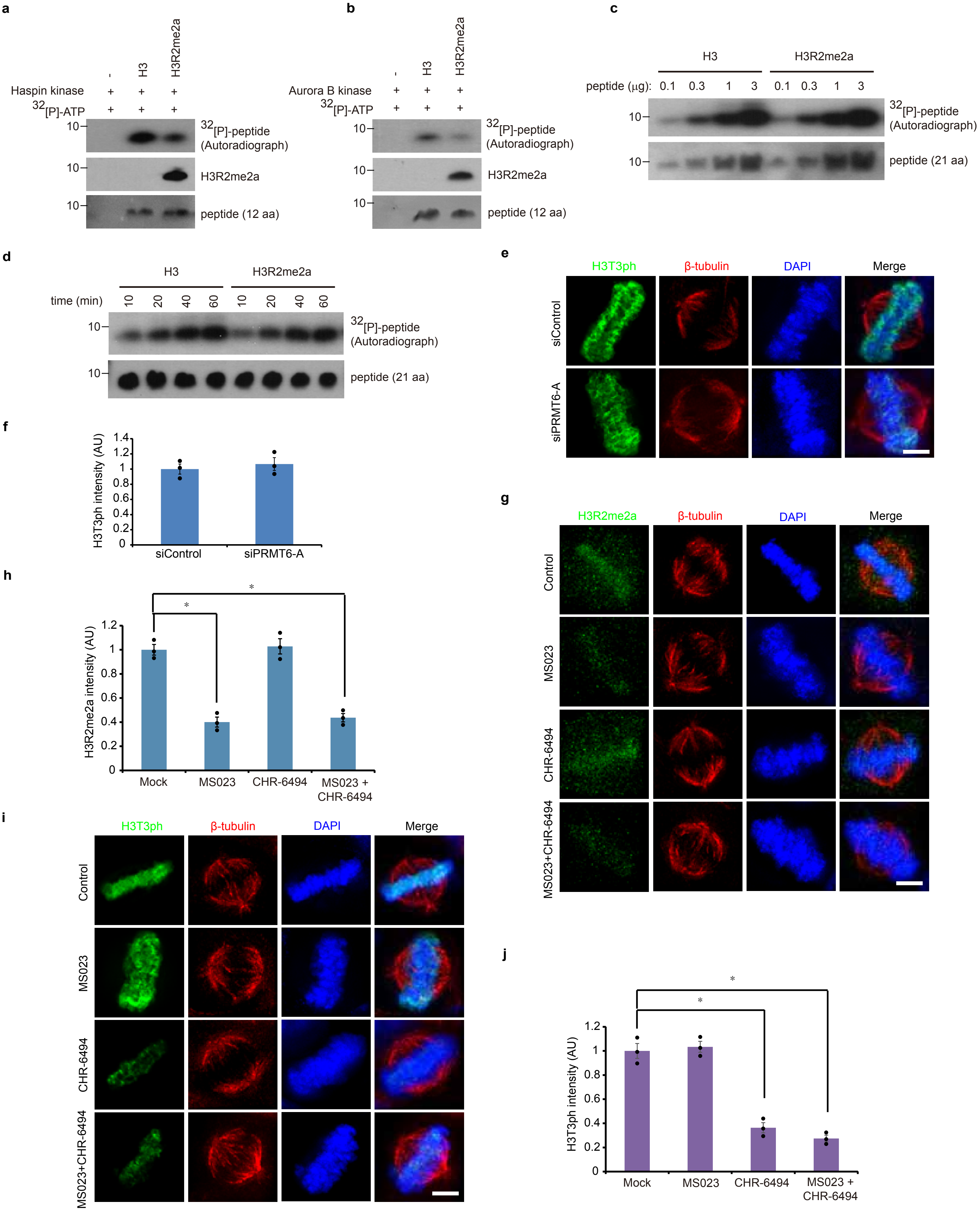

Supplementary Figure 10. H3R2 methylation and H3T3 phosphorylation are independent events. (a) *In vitro* Haspin kinase assay with the 12-aa peptide. **(b)** *In vitro* Aurora B kinase assay with the 12-aa peptide. **(c, d)** *In vitro* Aurora B kinase assay with the 21-aa peptide the assess phosphorylation kinetics. **(e, f)** The intensity of H3T3ph was analyzed by immunofluorescence microscopy in siRNA-transfected mitotic HeLa cells (n = 30 metaphase cells from three independent experiments). **(g-j)** The HeLa cells were treated with the indicated inhibitor and subjected to immunofluorescence staining. The images, acquired under a constant exposure time for all channels, are maximum projections from z stacks of representative cells stained for H3R2me2a or H3T3ph (green), β-tubulin (red), and DNA (blue). The intensities of H3R2me2a and H3T3ph were quantified and plotted (n = 30 metaphase cells from three independent experiments). Scale bars, 5 μm. Error bars, SEMs. Source data are provided as a Source Data file. (Student's t-test *$p < 0.01$).

a

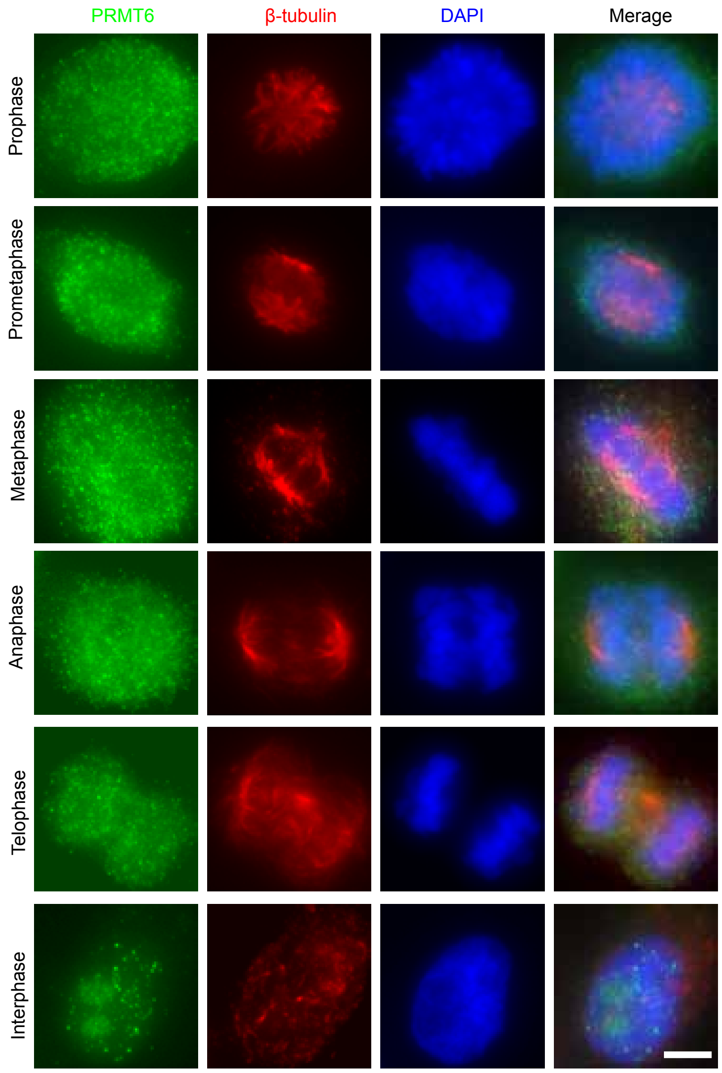

Supplementary Figure 11. The localization of PRMT6 during mitosis. (a) The images are maximum projections from z stacks of representative cells that were stained for PRMT6 (green), β-tubulin (red), and DNA (blue). Scale bar, 5 μm.

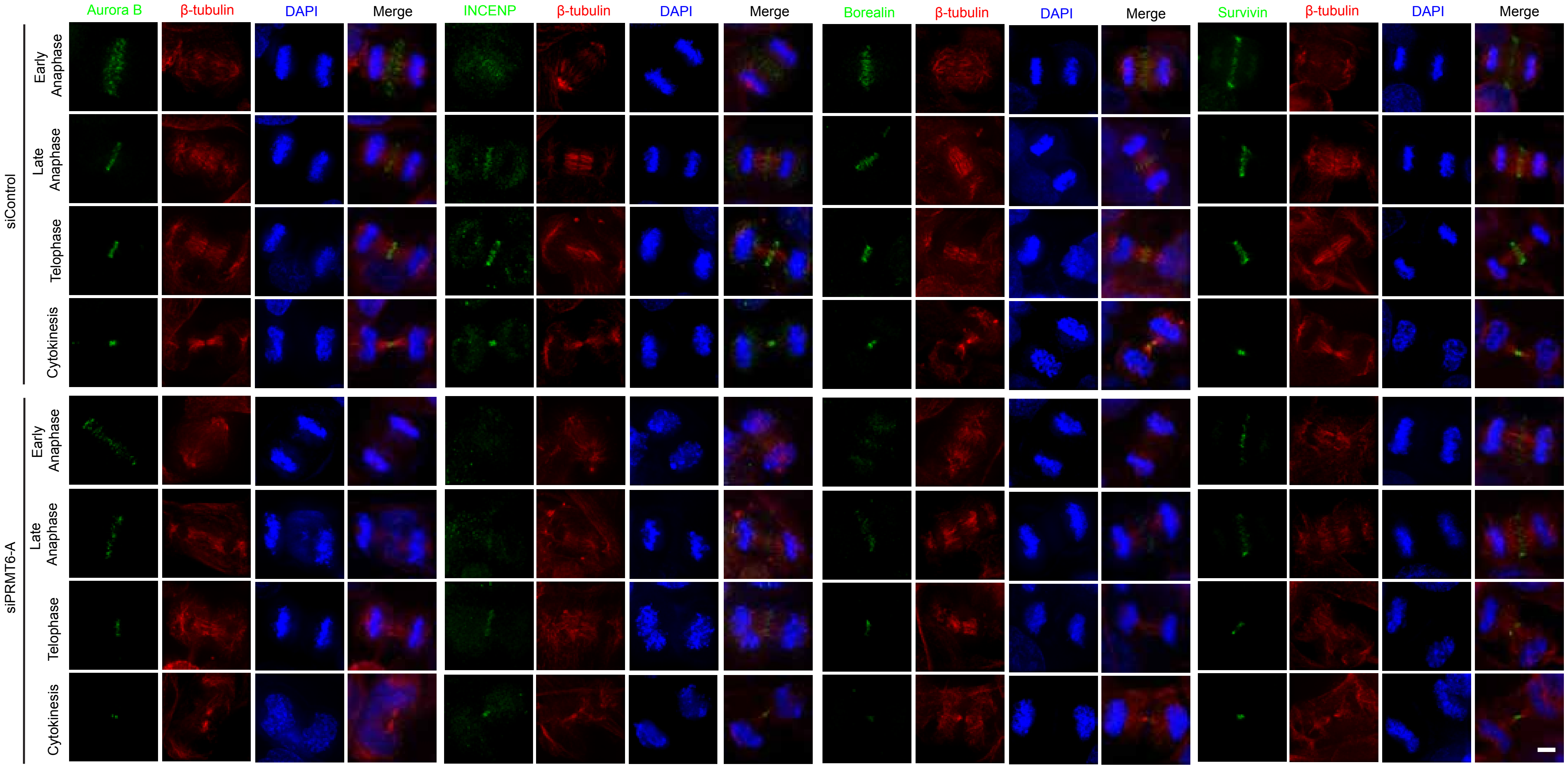

Supplementary Figure 12. CPC translocation to the central spindle and midbody in PRMT6-depleted cells. Immunofluorescence microscopy of CPC components during phases of late mitosis, including anaphase, telophase, and cytokinesis. The images are maximum projections from z stacks of representative cells that were stained for CPC (green), β-tubulin (red), and DNA (blue). Scale bar, 5 µm.

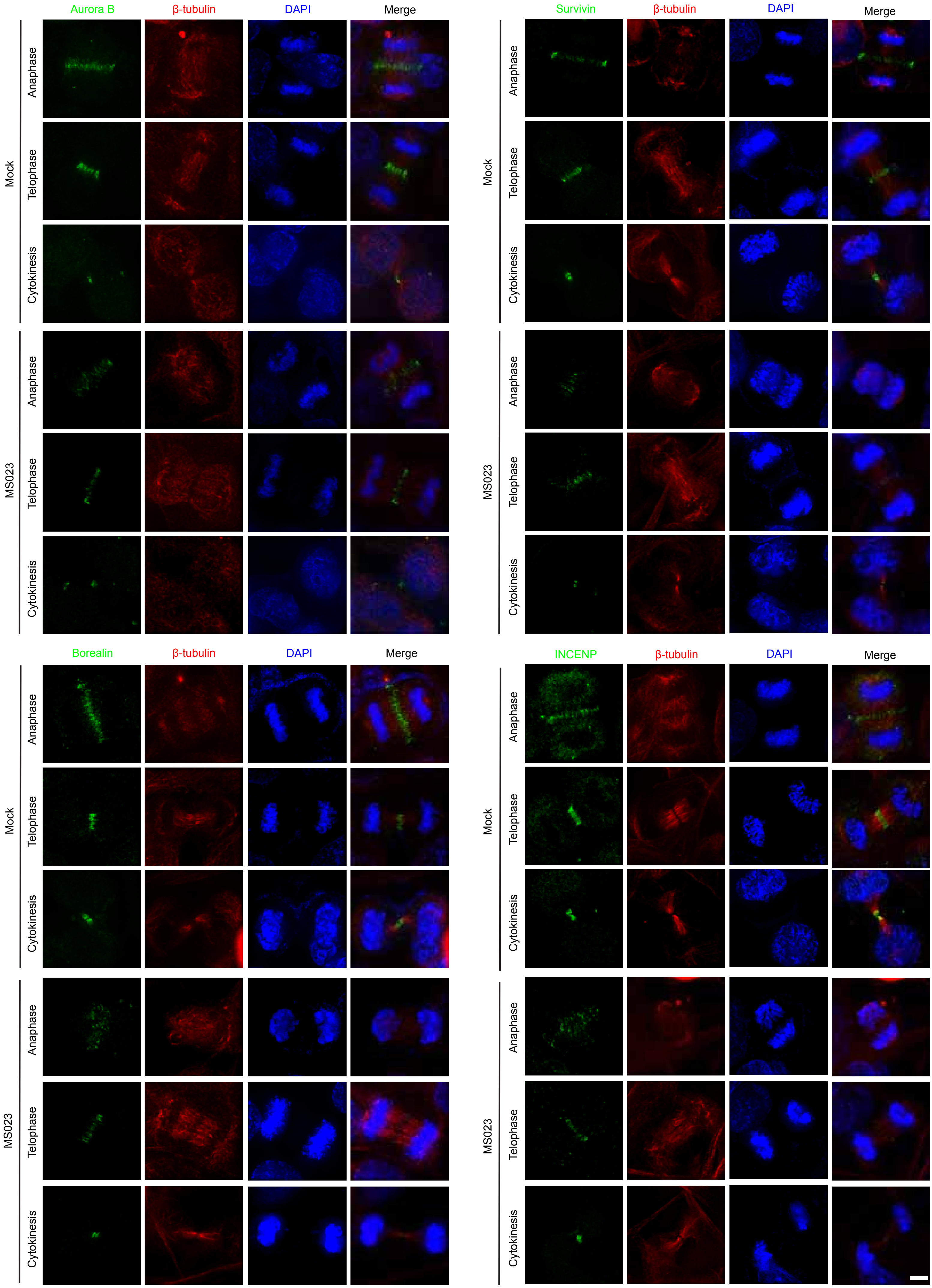

Supplementary Figure 13. CPC translocation to the central spindle and midbody in PRMT6-inhibited cells. Immunofluorescence microscopy of CPC components during phases of late mitosis, including anaphase, telophase, and cytokinesis. Scale bar, 5 μm.

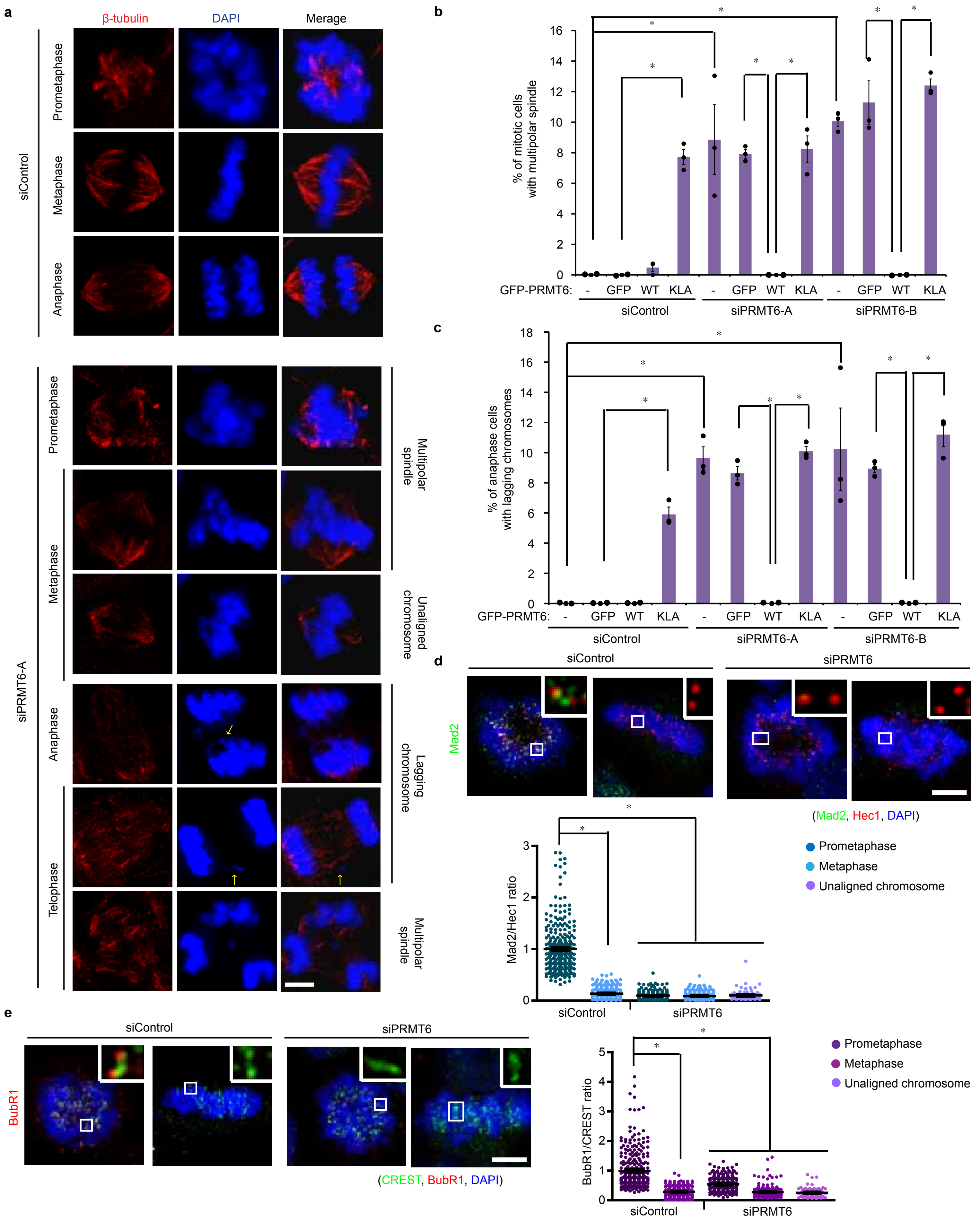

Supplementary Figure 14. PRMT6 depletion causes severe defects in mitotic progression. (a-c) Forty-four hours after siRNA transfection, HeLa cells were transfected with GFP-PRMT6 WT or mutant (KLA). Twenty-eight hours after DNA transfection, the cells were subjected to immunostaining for β-tubulin (red) and DNA (blue) and the number of mitotic cells with multipolar spindles from three independent experiments was quantified and plotted (b, n = 300 interphase cells). Anaphase cells with lagging chromosomes from three independent experiments were quantified and plotted (c, n = 100 anaphase cells). Arrows point to lagging chromosomes in the anaphase cells. **(d, e)** The maximum projections from z stacks of representative control or PRMT6-depleted cells stained for Mad2 or CREST (green), Hec1 or BubR1 (red), and DNA (blue) are shown. The Mad2/Hec1 ratio and BubR1/CREST ratio from three independent experiments were quantified and plotted (n = 300 kinetochores for prometaphase and metaphase; n = 90 kinetochores for unaligned chromosomes). Scale bars, 5 μm. Error bars, SEMs. P values were calculated by two-way ANOVA (b and c; *p < 0.01) or two-tailed Student's t-test (d and e; *p < 0.01). Source data are provided as a Source Data file.

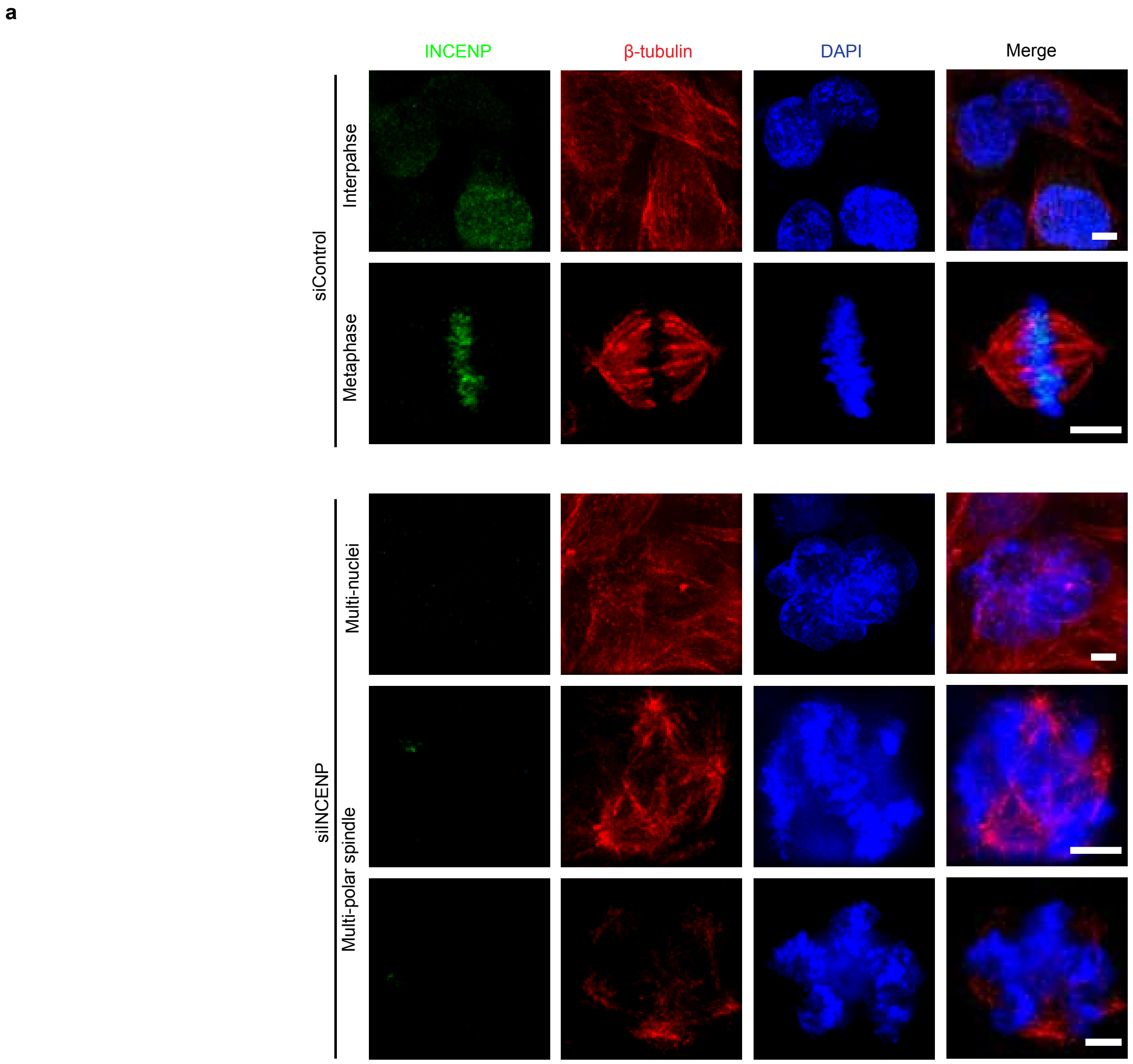

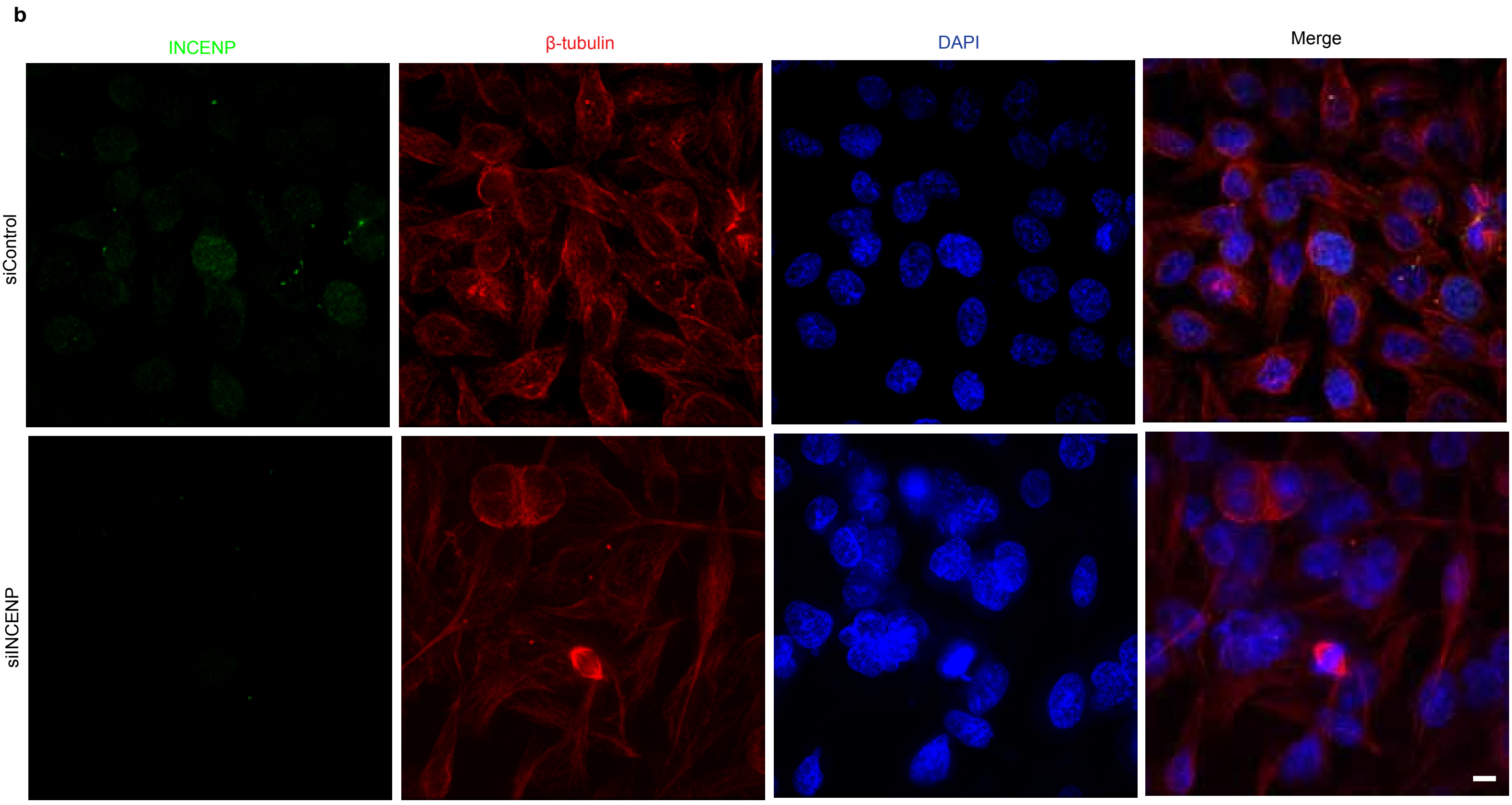

Supplementary Figure 15. Depletion of INCENP disrupts chromosome integrity. (a, b) HeLa cells were treated with siINCENP for 72 hours, fixed with MeOH, and stained with the indicated antibodies. The images are maximum projections from z stacks of representative cells stained for INCENP (green), β-tubulin (red), and DNA (blue). Scale bars, 5 μm.

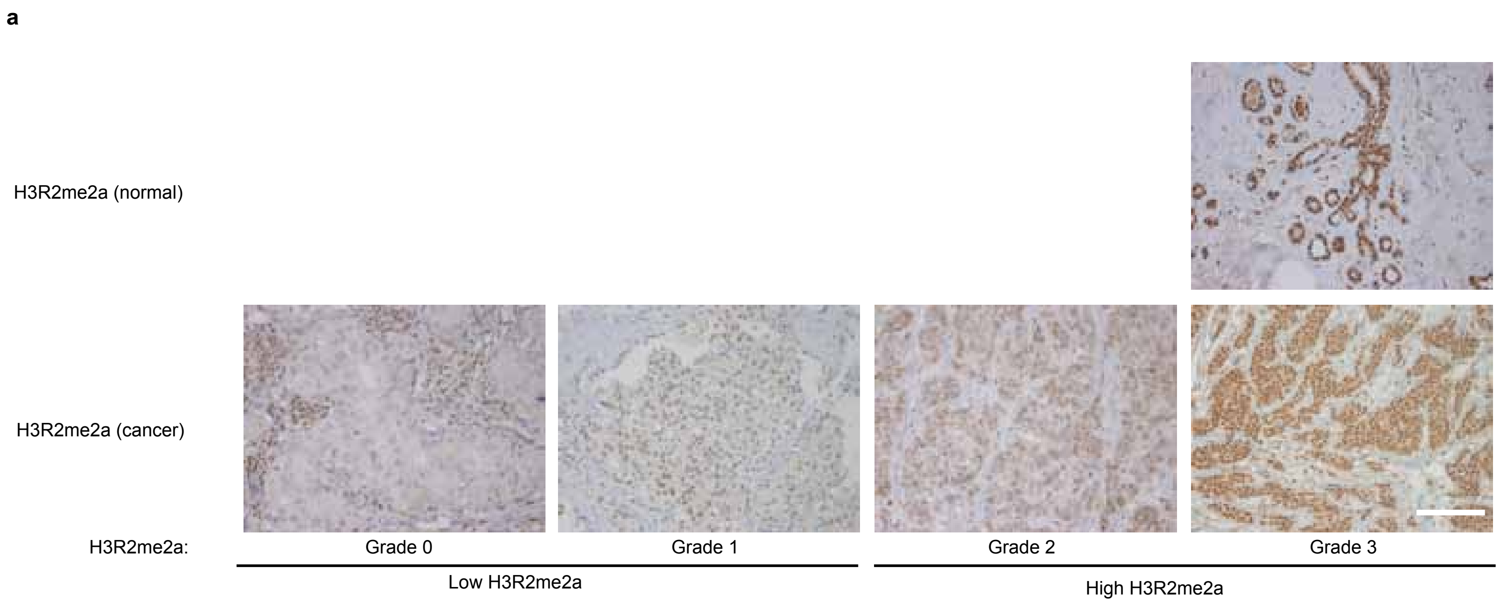

Supplementary Figure 16. The level of H3R2me2a is correlated with breast cancer. (a) Immunohistochemical staining of H3R2me2a in 410 human breast cancer patient tissues and normal adjacent tissues. Scale bar, 100 μm.

Supplementary Table 1. Primers used in this study

Primer name	Primer sequence
FLAG-HA-Histone H3 R2A-F	GAG TGA ACC ATG GCT GCT ACG AAG CAA ACA
FLAG-HA-Histone H3 R2A-R	TGT TTG CTT CGT AGC AGC CAT GGT TCA CTC
FLAG-HA-Histone H3 R2K-F	GAG TGA ACC ATG GCT AAG ACG AAG CAA ACA G
FLAG-HA-Histone H3 R2K-R	CTG TTT GCT TCG TCT TAG CCA TGG TTC ACT C
FLAG-HA-Histone H3 S10A-F	CAA ACA GCT CGC AAG GCT ACC GGC GGC AAA GC
FLAG-HA-Histone H3 S10A-R	GCT TTG CCG CCG GTA GCC TTG CGA GCT GTT TG
GFP-LacI-PRMT6-PCR-F	GGA ATT CAT GTC GCA GCC CAA GAA AAG AAA
GFP-LacI-PRMT6-PCR-R	GGA ATT CTC AGT CCT CCA TGG CAA AGT CTT
GFP-LacI-PRMT6-KLA-F	TCA ATT CTG CAG TCG ACG ATG TCG CAG CCC AAG AAA
GFP-LacI-PRMT6-KLA-R	TTT CTT GGG CTG CGA CAT CGT CGA CTG CAG AAT TGA
GFP-PRMT6 KLA-F	GAG GCA AGA CGA AAC TGG CCG TGG GCG CGG
GFP-PRMT6 KLA-R	CCG CGC CCA CGG CCA GTT TCG TCT TGC CTC
