# Supplementary material for: PRMT6-mediated H3R2me2a guides Aurora B to chromosome arms for proper chromosome segregation
Source: Nat Commun. 2020 Jan 30;11:612. doi: 10.1038/s41467-020-14511-w (PMC6992762; doi:10.1038/s41467-020-14511-w)
Supplement: Supplementary file 3 — Description of Additional Supplementary Files [file 41467_2020_14511_MOESM3_ESM.pdf]

Description of Additional Supplementary Files

File Name: Supplementary Movie 1
Description: **Fluorescence time-lapse microscopy of a control HeLa/GFP-H2B mitotic cell.** Images were acquired at 3-min intervals with a fluorescence microscope. The duration of mitotic progression was quantified and is presented in Supplementary Fig. 1e and f.

File Name: Supplementary Movie 2
Description: **Fluorescence time-lapse microscopy of a PRMT6-depleted HeLa/GFP-H2B mitotic cell.** Images were acquired at 3-min intervals with a fluorescence microscope. The duration of mitotic progression was quantified and is presented in Supplementary Fig. 1e and f.

File Name: Supplementary Movie 3
Description: **Tomographic time-lapse microscopy of a control HeLa mitotic cell.** Images were acquired at 1-min intervals with a tomographic microscope. The density of the metaphase chromosomes was quantified and is presented in Fig. 2b.

File Name: Supplementary Movie 4
Description: **Tomographic time-lapse microscopy of a PRMT6-depleted HeLa mitotic cell.** Images were acquired at 1-min intervals with a tomographic microscope. The density of the metaphase chromosomes was quantified and is presented in Fig. 2b.

File Name: Supplementary Movie 5
Description: **Fluorescence time-lapse microscopy of a control HeLa/GFP-CenpA prometaphase cell.** Images were acquired at 0.5-s intervals with a confocal microscope. The distance between CenpA pairs was quantified and is presented in Fig. 2g.

File Name: Supplementary Movie 6
Description: **Fluorescence time-lapse microscopy of a PRMT6-depleted HeLa/GFP-CenpA prometaphase cell.** Images were acquired at 0.5-s intervals with a confocal microscope. The distance between CenpA pairs was quantified and is presented in Fig. 2g.

File Name: Supplementary Movie 7
Description: **Fluorescence time-lapse microscopy of a control HeLa/GFP-CenpA metaphase cell.** Images were acquired at 0.5-s intervals with a confocal microscope. The distance between CenpA pairs was quantified and is presented in Fig. 2g.

File Name: Supplementary Movie 8
Description: **Fluorescence time-lapse microscopy of a PRMT6-depleted HeLa/GFP-CenpA metaphase cell.** Images were captured every 0.5 s with a confocal microscope. The distance between paired CenpA was quantified and is presented in Fig. 2g.

File Name: Supplementary Movie 9
Description: **Fluorescence time-lapse microscopy of a PRMT6 inhibitor-treated HeLa/GFP-CenpA prometaphase cell.** Images were acquired at 0.5-s intervals with a confocal microscope. The distance between CenpA pairs was quantified and is presented in Supplementary Fig. 4e.

File Name: Supplementary Movie 10
Description: **Fluorescence time-lapse microscopy of a PRMT6 inhibitor-treated HeLa/GFP-CenpA metaphase cell.** Images were acquired at 0.5-s intervals with a confocal microscope. The distance between CenpA pairs was quantified and is presented in Supplementary Fig. 4e.
