# Supplementary material for: PRMT6-mediated H3R2me2a guides Aurora B to chromosome arms for proper chromosome segregation
Source: Nat Commun. 2020 Jan 30;11:612. doi: 10.1038/s41467-020-14511-w (PMC6992762; doi:10.1038/s41467-020-14511-w)
Supplement: Supplementary file 14 — Reporting Summary [file 41467_2020_14511_MOESM14_ESM.pdf]

natureresearch

Corresponding author(s): DBPR NCOMMS-18-22563

Last updated by author(s): Feb 27, 2019

Reporting Summary

Nature Research wishes to improve the reproducibility of the work that we publish. This form provides structure for consistency and transparency in reporting. For further information on Nature Research policies, see Authors & Referees and the Editorial Policy Checklist.

Statistics

For all statistical analyses, confirm that the following items are present in the figure legend, table legend, main text, or Methods section.

n/a	Confirmed	
☐	☒	The exact sample size (*n*) for each experimental group/condition, given as a discrete number and unit of measurement
☐	☒	A statement on whether measurements were taken from distinct samples or whether the same sample was measured repeatedly
☐	☒	The statistical test(s) used AND whether they are one- or two-sided<br>*Only common tests should be described solely by name; describe more complex techniques in the Methods section.*
☐	☒	A description of all covariates tested
☐	☒	A description of any assumptions or corrections, such as tests of normality and adjustment for multiple comparisons
☐	☒	A full description of the statistical parameters including central tendency (e.g. means) or other basic estimates (e.g. regression coefficient) AND variation (e.g. standard deviation) or associated estimates of uncertainty (e.g. confidence intervals)
☐	☒	For null hypothesis testing, the test statistic (e.g. *F*, *t*, *r*) with confidence intervals, effect sizes, degrees of freedom and *P* value noted<br>*Give P values as exact values whenever suitable.*
☒	☐	For Bayesian analysis, information on the choice of priors and Markov chain Monte Carlo settings
☒	☐	For hierarchical and complex designs, identification of the appropriate level for tests and full reporting of outcomes
☒	☐	Estimates of effect sizes (e.g. Cohen's *d*, Pearson's *r*), indicating how they were calculated

Our web collection on statistics for biologists contains articles on many of the points above.

Software and code

Policy information about availability of computer code

Data collection	No software was used.
Data analysis	GraphPad Prism software (version 5)

For manuscripts utilizing custom algorithms or software that are central to the research but not yet described in published literature, software must be made available to editors/reviewers. We strongly encourage code deposition in a community repository (e.g. GitHub). See the Nature Research guidelines for submitting code & software for further information.

Data

Policy information about availability of data

All manuscripts must include a data availability statement. This statement should provide the following information, where applicable:

- Accession codes, unique identifiers, or web links for publicly available datasets
- A list of figures that have associated raw data
- A description of any restrictions on data availability

All relevant data are readily available from the authors.

Field-specific reporting

Please select the one below that is the best fit for your research. If you are not sure, read the appropriate sections before making your selection.

☒ Life sciences ☐ Behavioural & social sciences ☐ Ecological, evolutionary & environmental sciences

For a reference copy of the document with all sections, see nature.com/documents/nr-reporting-summary-flat.pdf

Life sciences study design

All studies must disclose on these points even when the disclosure is negative.

Sample size	For fluorescence intensity in mitotic cell, we measured 30 cells from three independent experiments. Because the percentage of mitotic cells in culture dish is about 1~2%, 10 cells are sufficient for each experiment. For CPC intensity, we measured more than 100 centromeres and chromosome arms from three independent experiments. For live cell image, individual data points are shown when the number of data points is <10.
Data exclusions	No data were excluded from the analyses.
Replication	All attempts at replication were successful.
Randomization	All samples were randomly allocated into experimental group.
Blinding	For the blind setup, we masked the name of the samples on slide glasses and the quantifications were performed by a different person who did not perform the experiment.

Reporting for specific materials, systems and methods

We require information from authors about some types of materials, experimental systems and methods used in many studies. Here, indicate whether each material, system or method listed is relevant to your study. If you are not sure if a list item applies to your research, read the appropriate section before selecting a response.

Materials & experimental systems

n/a	Involved in the study	
☐	☒	Antibodies
☐	☒	Eukaryotic cell lines
☒	☐	Palaeontology
☒	☐	Animals and other organisms
☒	☐	Human research participants
☐	☒	Clinical data

Methods

n/a	Involved in the study	
☒	☐	ChIP-seq
☐	☒	Flow cytometry
☒	☐	MRI-based neuroimaging

Antibodies

Antibodies used

Format:
Antibody name (Species, Applications, Cat. NO., LOT. NO., Clone name/number, Company)

[Primary antibody]
anti-Flag (mouse, WB/IF/ICC/EIA, F1804, SLBK1346V, M2, Sigma Inc.)
anti-b-actin (mouse, WB/IF, A5441, -, AC-15, Sigma Inc.)
anti-a-tubulin (mouse, WB/ICC/IP/ARR, T6074, -, B-5-1-2, Sigma Inc.)
anti-H3T3ph (rabbit, FC/ICC/IP/WB/IHC-P, ab78351,GR149862-1, EP1702Y, Abcam)
anti-H3S10ph (rabbit, ICC/IF/WB/IHC/IP/IHC-P/ELISA, ab32107, -, E173, Abcam)
anti-INCENP (rabbit, ICC/IF/IHC/IP/WB, ab12183, GR166659-1, -, Abcam)
anti-Survivin (rabbit, FC/ICC/IF/IP/WB/Sandwich ELISA/IHC-P, ab76424, GR 309461-3, EP2880Y, Abcam)
anti-H4K16ac (rabbit, WB/IHC-P/ICC/IF/FC, ab109463, -, EPR1004, Abcam)
anti-H3S28ph (rabbit, WB/IHC-P/DB/ICC/IF/FC/ChIP/IHC-FoFr, ab32388, -, E191, Abcam)
anti-Hsp90 (mouse, WB/IP/ELISA, sc-69703, D0315, 4F10, Santa Cruz Biotechnology, Inc.)
anti-Aurora B (rabbit, WB/IP/IF/ELISA, sc-25426, D2811, H-75, Santa Cruz Biotechnology, Inc.)
anti-p38 MAPK (rabbit, WB/IP/IF/IHC-P, sc-728, -, N-20, Santa Cruz Biotechnology, Inc.)
anti-Cyclin A (mouse, WB/IP/IHC/IF/ELISA/FC, sc-271682, C2613, B-8, Santa Cruz Biotechnology, Inc.)
anti-Cyclin B1 (rabbit, WB, sc-594, -, H-20,Santa Cruz Biotechnology, Inc.)
anti-Cyclin E (mouse, WB/IP/IF/IHC, sc-247, I1214, HE12, Santa Cruz Biotechnology, Inc.)
anti-H3R2me2a (rabbit, WB/PIA, 07-585, 2864357, -, Merck Millipore)
anti-HA (rabbit or goat, WB/IP/IF/FCM/ELISA, sc-805, -, Y-11, Santa Cruz Biotechnology, Inc.)
anti-H3K9me3(rabbit, DB/ ICC/ PIA, 07-442, -, - Merck Millipore)
anti-Histone H4 (rabbit, WB, 07-108, -, -, Merck Millipore)
anti-H3R17me2a (rabbit, ELISA/ICC/WB/PIA, 07-214, -, -, Merck Millipore)
anti-H3K4me3 (rabbit, ChIP/ChIP-Seq/DB/ICC/IF/WB, 39159, -, -, Active motif)
anti-Hec1(mouse, FACS/ICC/IHC-fr/IF/IP/WB/PLA, GTX70268, -, 9G3.23, GeneTex, Inc.)
anti-Mad2 (rabbit, IP/IF/IHC-P/WB/ICC, PA5-21594, -, -, Thermo Scientific Pierce Antibodies);
anti-Shugoshin (rabbit, ICC/IF/WB, PA5-30869, -, -, Thermo Scientific Pierce Antibodies)
anti-b-tubulin E7 monoclonal antibody (mouse, IF/IHC/IP/WB, E7, -, -, Developmental Studies Hybridoma Bank, USA)
anti-Borealin (rabbit, WB/ICC/IF/IHC/IHC-P, NBP1-89951, -, -, NOVUS);
anti-CREST (human, IF, 15-235-0001, -, -, antibodies Incorporated);

anti-PRMT6 (rabbit, WB/IHC-P/IF, A300-828A, A300-928A-1, -, BETHYL);
anti-BubR1 (mouse, WB/IF, LS-C2771, 47929, 8G1, LifeSpan BioSciences);
anti-Histone H3 (rabbit, WB/IP/IF/IHC-P, 9715, -, -, Cell Signaling),
anti-H3K9ac (rabbit, WB/IP/IF/IHC/ChIP/FC, 9649, -, C5B11, Cell Signaling),
anti-H3S10ph (rabbit, WB/IHC-P,F/IF/ICC/FC, 9701, -, -, Cell Signaling).
anti-HP1 (rabbit, WB/IP/IHC-P/IF-IC/ChIP/FC, 2616, -, -, Cell signaling)
anti-SGO2 (rabbit, IHC/ICC-IF, HPA035163, D118712, SGO2, ATLAS)
anti-Bub1 (rabbit, IP/IF/ICC, MA1-5755, UG2806321, 14H5, Invitrogen)
Anti-histone H2A T120ph (rabbit, DB/WB, 39391, 30508001, Histone H2AT120ph, active motif)
Anti-CENP-C (rabbit, WB/IP/IHC/ICC, PDO30, 006, CENP-C, MBL international corporation)

[Secondary antibody]
CST Anti-rabbit IgG, HRP-linked Antibody (goat, WB, 7074S, -,-, Cell Signaling Technology Inc)
Mouse IgG antibody (HRP) (goat, WB/ICC/IF/IHC-P/ELISA/IHC, GTX213111-01, -,-, Gene Tex Inc)
Alexa Fluor® 488 goat anti-rabbit IgG (goat, FC/ICC/IF/IHC/IHC(P)/MISC, A11034, -, -, Thermo Scientific Pierce Antibodies)
Alexa Fluor® 594 goat anti-mouse IgG (goat, FC/ICC/IF/IHC/ICC/IHC(P)/MISC, A11032, -, -, Thermo Scientific Pierce Antibodies)
Alexa Fluor® 594 goat anti-rabbit IgG (goat, FC/ICC/IF/IHC/IHC(P)/ICC/MISC, A11037, -, -, Thermo Scientific Pierce Antibodies)
Alexa Fluor® 488 goat anti-human IgG (goat, FC/IF/ICC/IHC/IHC(F)/MISC, A11013, -, -, Thermo Scientific Pierce Antibodies)

Validation

Format:
Antibody name (Species, Applications, Cat. NO., LOT. NO., Clone name/number, Company)

[Primary antibody]

anti-Flag (mouse, WB/IF/ICC/EIA, F1804, SLBK1346V, M2, Sigma Inc.)
Species reactivity: Mammalian
Application: WB/EIA/IP
Citation: Bjerrum, O.J., and Heegaard. N.H.H., CRC Handbook of Immunoblotting of Proteins, Volume I, Technical Descriptions, CRC Press (Boca Raton, FL: 1988) 229-236.

anti-b-actin (mouse, WB/IF, A5441, -, AC-15, Sigma Inc.)
Species reactivity: Guinea pig, Canine, Hirudo medicinalis, Feline, Pig, Carp, Mouse, Chicken, Rabbit, Sheep, Rat, Human, Bovine
Application: ELISA/IF/IHC/WB
Citation: Lessard, J., Two monoclonal antibodies to actin: one muscle selective and one generally reactive Cell Motil. Cytoskel. 10, 349 (1988).

anti-a-tubulin (mouse, WB/ICC/IP/ARR, T6074, -, B-5-1-2, Sigma Inc.)
Species reactivity: Human, Chlamydomonas, African green monkey, Chicken, Kangaroo rat, Bovine, Mouse, Rat, Sea urchin
Application: ARR/ICC/IP/WB
Citation: Piperno G et al., Microtubules containing acetylated alpha-tubulin in mammalian cells in culture, J. Cell Biol., 104, 289-302 (1987).

anti-H3T3ph (rabbit, FC/ICC/IP/WB/IHC-P, ab78351,GR149862-1, EP1702Y, Abcam)
Species reactivity: Human
Application: FC/WB/IHC-P/ICC
Citation: Lanza C et al. Reduced O-GlcNAcase expression promotes mitotic errors and spindle defects. Cell Cycle 15:1363-75 (2016).

anti-H3S10ph (rabbit, ICC/IF/WB/IHC/IP/IHC-P/ELISA, ab32107, -, E173, Abcam)
Species reactivity: Mouse, Rat, Human, Drosophila melanogaster
Application: ELISA/DB/ICC/IF/WB/IHC-P/IP
Citation: Fiskus W et al. Cotreatment with vorinostat enhances activity of MK-0457 (VX-680) against acute and chronic myelogenous leukemia cells. Clin Cancer Res 14:6106-15 (2008).

anti-INCENP (rabbit, ICC/IF/IHC/IP/WB, ab12183, GR166659-1, -, Abcam)
Species reactivity: Mouse, Rat, Human, Xenopus laevis
Application: WB/ICC/IF/IHC-Fr/IP
Citation: Vader G et al. Survivin mediates targeting of the chromosomal passenger complex to the centromere and midbody. EMBO Rep 7:85-92 (2006).

anti-Survivin (rabbit, FC/ICC/IF/IP/WB/Sandwich ELISA/IHC-P, ab76424, GR 309461-3, EP2880Y, Abcam)
Species reactivity: Human
Application: IHC-P/WB/IP/FC/ICC/IF/Sandwich ELISA
Citation: Manuel ER et al. Enhancement of cancer vaccine therapy by systemic delivery of a tumor-targeting Salmonella-based STAT3 shRNA suppresses the growth of established melanoma tumors. Cancer Res 71:4183-91 (2011).

anti-H4K16ac (rabbit, WB/IHC-P/ICC/IF/FC, ab109463, -, EPR1004, Abcam)
Species reactivity: Human, Mouse, Rat
Application: WB/IHC-P/ICC/IF/FC
Citation: Marcos-Villar L et al., Epigenetic control of influenza virus: role of H3K79 methylation in interferon-induced antiviral response. Sci Rep 8(1):1230 (2018).

anti-H3S28ph (rabbit, WB/IHC-P/DB/ICC/IF/FC/ChIP/IHC-FoFr, ab32388, -, E191, Abcam)
Species reactivity: Mouse, Rat, Guinea pig, Human
Application: WB/IHC-P/DB/ICC/IF/FC/ChIP/IHC-FoFr
Citation: Xiong K et al. Layer I as a putative neurogenic niche in young adult guinea pig cerebrum. Mol Cell Neurosci 45:180-91 (2010).

anti-Hsp90 (mouse, WB/IP/ELISA, sc-69703, D0315, 4F10, Santa Cruz Biotechnology, Inc.)
Species reactivity: Human
Application: WB/IP/ELISA
Citation: Kimura T et al., Expression of Bcl-2 and Bax in Hypokalemic Nephropathy in Rats. Pathobiology 69:237–248 (2001)

anti-Aurora B (rabbit, WB/IP/IF/ELISA, sc-25426, D2811, H-75, Santa Cruz Biotechnology, Inc.)
Species reactivity: Human, Bovine, Porcine
Application: WB/IP/IF/ELISA
Citation: Uzbekova S et al., Spatio-temporal expression patterns of aurora kinases a, B, and C and cytoplasmic polyadenylation-element-binding protein in bovine oocytes during meiotic maturation. Biol Reprod 78(2):218-33 (2008)

anti-p38 MAPK (rabbit, WB/IP/IF/IHC-P, sc-728, -, N-20, Santa Cruz Biotechnology, Inc.)
Species reactivity: Human, Mouse, Rat, Avian, Canine, Bovine, Porcine
Application: WB/IP/IF/IHC(P)
Citation: Shimizu N et al., Activation of mitogen-activated protein kinases and activator protein-1 in myocardial infarction in rats. Cardiovasc Res 38(1):116-24 (1998)

anti-Cyclin A (mouse, WB/IP/IHC/IF/ELISA/FC, sc-271682, C2613, B-8, Santa Cruz Biotechnology, Inc.)
Species reactivity: Human
Application: WB/IP/IF/IHC/ELISA
Citation: Xu T et al., ECRG4 inhibits growth and invasiveness of squamous cell carcinoma of the head and neck in vitro and in vivo. Oncol Lett 5(6):1921–1926 (2013)

anti-Cyclin B1 (rabbit, WB, sc-594, -, H-20,Santa Cruz Biotechnology, Inc.)
Species reactivity: Human, Equine, Canine, Bovine, Porcine
Application: WB/IP/IF/ELISA
Citation: Munro J et al., Human fibroblast replicative senescence can occur in the absence of extensive cell division and short telomeres. Oncogene 20(27):3541-52 (2001)

anti-Cyclin E (mouse, WB/IP/IF/IHC, sc-247, I1214, HE12, Santa Cruz Biotechnology, Inc.)
Species reactivity: Human, Mouse, Rat
Application: WB/IP/IF/IHC(P)
Citation: Oishi T et al., Galectin-3 may contribute to Cisplatin resistance in clear cell carcinoma of the ovary. Int J Gynecol Cancer 17(5):1040-6 (2007)

anti-HA (rabbit or goat, WB/IP/IF/FCM/ELISA, sc-805, -, Y-11, Santa Cruz Biotechnology, Inc.)
Species reactivity: influenza hemagglutinin (HA) protein
Application: WB/IP/IF/FCM/ELISA
Citation: Fuller SJ et al., A novel non-canonical mechanism of regulation of MST3 (mammalian Sterile20-related kinase 3). Biochem J 442(3):595-610 (2012)

anti-H3R2me2a (rabbit, WB/PIA, 07-585, 2864357, -, Merck Millipore)
Species reactivity: Human, Chicken
Application: WB/PIA
Citation: Hyllus D et al., PRMT6-mediated methylation of R2 in histone H3 antagonizes H3 K4 trimethylation. UM Genes & development 21(24): 3369–3380

anti-H3K9me3(rabbit, DB/ ICC/ PIA, 07-442, -, - Merck Millipore)
Species reactivity: Human, Mouse, Rat, Chicken
Application: DB/ICC/Mplex/PIA/WB/ChiP-seq
Citation: Ma Y et al., DNA CpG hypomethylation induces heterochromatin reorganization involving the histone variant macroH2A. J Cell Sci 118(Pt 8):1607-16 (2005)

anti-Histone H4 (rabbit, WB, 07-108, -, -, Merck Millipore)
Species reactivity: Human, Mouse, Bovine, Avian, Chicken, Xenopus
Application: WB
Citation: Gilbert S L et al. XIST RNA associates with specific regions of the inactive X chromatin. J. Biol. Chem 275: 36491-4 (2000)

anti-H3R17me2a (rabbit, ELISA/ICC/WB/PIA, 07-214, -, -, Merck Millipore)
Species reactivity: Human, Vertebrate
Application: ELISA/ICC/WB/PIA
Citation: Chadwick BP et al. Chromatin of the Barr body: histone and non-histone proteins associated with or excluded from the inactive X chromosome. Hum Mol Genet 1;12(17):2167-78.(2003)

anti-H3K4me3 (rabbit, ChIP/ChIP-Seq/DB/ICC/IF/WB, 39159, -, -, Active motif)
Species reactivity: Budding Yeast, Human, Mouse
Application: ChIP/ChIP-Seq/WB/IF/DB/IHC/ICC/FC
Citation: Gomez-Rodriguez J et al., Differential expression of interleukin-17A and -17F is coupled to T cell receptor signaling via inducible T cell kinase. Immunity 31(4):587-97 (2009)

anti-Hec1(mouse, FACS/ICC/IHC-fr/IF/IP/WB/PLA, GTX70268, -, 9G3.23, GeneTex, Inc.)
Species reactivity: Green monkey, Kangaroo rat, Human, Golden Syrian hamster, House mouse, Rat, Pig
Application: FC/FACS/ICC-IF/IHC/IP/PLA/WB
Citation: Zhu H, Coppinger J, Jang C, Yates J, Fang G. FAM29A promotes microtubule amplification via recruitment of the NEDD1-gamma-tubulin complex to the mitotic spindle. J Cell Biol. 2008;183:835-48

anti-Mad2 (rabbit, IP/IF/IHC-P/WB/ICC, PA5-21594, -, -, Thermo Scientific Pierce Antibodies);
Species reactivity: Human
Application: ICC/IF/IFC(P)/IP/WB
Citation: Choi M et al., Polo-like kinase 1 inhibitor BI2536 causes mitotic catastrophe following activation of the spindle assembly checkpoint in non-small cell lung cancer cells. Cancer Lett 357(2):591-601 (2015)

anti-Shugoshin (rabbit, ICC/IF/WB, PA5-30869, -, -, Thermo Scientific Pierce Antibodies)
Species reactivity: Human
Application: ICC/IF/WB
Citation: Agircan FG, Schiebel E, Sensors at centrosomes reveal determinants of local separase activity. PLos Genet 10(10):e1004672 (2014)

anti-b-tubulin E7 monoclonal antibody (mouse, IF/IHC/IP/WB, E7, -, -, Developmental Studies Hybridoma Bank, USA)
Species Reactivity: Chlamydomonas, Drosophila, Flatworm, Giant panda, Human, Kangaroo Rat, Mouse, Xenopus
Recommended applications: IF/IHC/IP/WB
Citation: Chu DT, Klymkowsky MW, The appearance of acetylated alpha-tubulin during early development and cellular differentiation in Xenopus. Dev Biol 136(1):104-17 (1989)

anti-Borealin (rabbit, WB/ICC/IF/IHC/IHC-P, NBP1-89951, -, -, NOVUS);
Species reactivity: Human
Application: WB/ICC/IF/IHC/IHC(P)
Citation: Figure 5K in this manuscript.

anti-CREST (human, IF, 15-235-0001, -, -, antibodies Incorporated);
Species reactivity: Human
Application: ICC
Citation: Jang CY et al., DDA3 Recruit Microtubule depolymerase Kif2a to Spindle Poles and Controls Spindle Dynamics and Mitotic Chromosome Movement. J. Cell Biol. 181 (2), 255-267 (2008)

anti-PRMT6 (rabbit, WB/IHC-P/IF, A300-828A, A300-928A-1, -, BETHYL);
Species reactivity: Human
Application: WB/IP/IHC/ICC
Citation: Iberg, A. N., Espejo, A., et al., Arginine methylation of the histone H3 tail impedes effector binding. Journal of Biological Chemistry 283 (6) 3006-3010 (2008)

anti-BubR1 (mouse, WB/IF, LS-C2771, 47929, 8G1, LifeSpan BioSciences);
Species reactivity: Human, Mouse
Application: IF/WB
Citation: Kwon HJ et al., DDA3 and Mdp3 modulate Kif2a recruitment onto the mitotic spindle to control minus-end spindle dynamics. Journal of cell science 129:2719-25 (2016)

anti-Histone H3 (rabbit, WB/IP/IF/IHC-P, 9715, -, -, Cell Signaling),
Species reactivity: Human, Mouse, Rat, Monkey, Zebrafish, Pig
Application: WB
Citation: Thorne AW et al., Patterns of histone acetylation. Eur J Biochem 13;193(3):701-13 (1990)

anti-H3K9ac (rabbit, WB/IP/IF/IHC/ChIP/FC, 9649, -, C5B11, Cell Signaling),
Species reactivity: Human, Mouse, Rat, Monkey, Zebrafish, C. elegans
Application: WB/IP/IHC-P/IF-IC/ChIP/ChIP-seq/FC Citation: Thorne AW et al., Patterns of histone acetylation. Eur J Biochem 13;193(3):701-13 (1990)

anti-H3S10ph (rabbit, WB/IHC-P,F/IF/ICC/FC, 9701, -, -, Cell Signaling).
Species reactivity: Human, Mouse, Rat, Monkey, S. cerevisiae, D. melanogaster
Application: WB/IHC-P/IHC-F/IF-IC/FC
Citation: Thorne AW et al., Patterns of histone acetylation. Eur J Biochem 13;193(3):701-13 (1990)

anti-HP1 (rabbit, WB/IP/IHC-P/IF-IC/ChIP/FC, 2616, -, -, Cell signaling)
Species reactivity: Human, Mouse, Rat, Monkey
Application: WB/IP/IHC-P/IF-IC/ChIP/FC
Citation: Minc E et al., Localization and phosphorylation of HP1 proteins during the cell cycle in mammalian cells. Chromosoma 108(4):220-34 (1999)

anti-SGO2 (rabbit, IHC/ICC-IF, HPA035163, D118712, SGO2, ATLAS)
Species reactivity: human
Application: IHC/ICC-IF
Citation: Lindsey SF et al., Potential role of meiosis proteins in melanoma chromosomal instability. J Skin Cancer 2013:190109, 9 pages (2013)

anti-Bub1 (rabbit, IP/IF/ICC, MA1-5755, UG2806321, 14H5, Invitrogen)
Species reactivity: human
Application: IP/IF/ICC
Citation: https://www.thermofisher.com/antibody/product/Bub1-Antibody-clone-14H5-Monoclonal/MA1-5755

Anti-histone H2A T120ph (rabbit, DB/WB, 39391, 30508001, Histone H2AT120ph, active motif)
Species reactivity: human, Wide range predicted
Application: DB/WB
Citation: Ciossanim G., Overlack, K., et al., The kinetochore proteins CENP-E and CENP-F directly and specifically interact with distinct BUB mitotic checkpoint Ser/Thr kinase. Journal of Biological Chemistry 293 (26) 10084-10101(2018)

Anti-CENP-C (rabbit, WB/IP/IHC/ICC, PDO30, 006, CENP-C, MBL international corporation)
Species reactivity: human
Application: WB/IP/IHC/ICC
Citation: Kuijt TE et al., Conditional targeting of MAD1 to kinetochores is sufficient to reactivate the spindle assembly checkpoint in metaphase. Chromosoma 123(5):471-80 (2014)

[Secondary antibody]

CST Anti-rabbit IgG, HRP-linked Antibody (goat, WB, 7074S, -,-, Cell Signaling Technology Inc)
Species reactivity: Oryctolagus cuniculus (Rabbit)
Application: WB
Citation: Imamura M et al. Transcriptional repression and DNA hypermethylation of a small set of ES cell marker genes in male germline stem cells. BMC Developmental Biology 21;6:34 (2006).

Mouse IgG antibody (HRP) (goat, WB/ICC/IF/IHC-P/ELISA/IHC, GTX213111-01, -,-, Gene Tex Inc)
Species reactivity: Mouse
Application: WB/ICC/IF/IHC-P/ELISA/IHC
Citation: Xie H et al. Functional studies of MLC1 mutations in Chinese patients with megalencephalic leukoencephalopathy with subcortical cysts. PLoS One 7 (3):e33087 (2012)

Alexa Fluor® 488 goat anti-rabbit IgG (goat, FC/ICC/IF/IHC/IHC(P)/MISC, A11034, -, -, Thermo Scientific Pierce Antibodies)
Species reactivity: Rabbit
Application: FC/ICC/IF/IHC/IHC(P)/MISC
Citation: Lederer CW et al., Pathways and genes differentially expressed in the motor cortex of patients with sporadic amyotrophic lateral sclerosis. BMC genomics 8:26 (2007)

Alexa Fluor® 594 goat anti-mouse IgG (goat, FC/ICC/IF/IHC/ICC/IHC(P)/MISC, A11032, -, -, Thermo Scientific Pierce Antibodies)
Species reactivity: Mouse
Application: FC/ICC/IF/IHC/ICC/IHC(P)/MISC
Citation: Franz CK et al., Intraspinal cord delivery of IGF-I mediated by adeno-associated virus 2 is neuroprotective in a rat model of familial ALS. Neurobiology of disease 33(3):473-81 (2009)

Alexa Fluor® 594 goat anti-rabbit IgG (goat, FC/ICC/IF/IHC/IHC(P)/ICC/MISC, A11037, -, -, Thermo Scientific Pierce Antibodies)
Species reactivity: Rabbit
Application: FC/ICC/IF/IHC/IHC(P)/ICC/MISC
Citation: Cushman-Nick M et al., Hsp104 suppresses polyglutamine-induced degeneration post onset in a drosophila MJD/SCA3 model. PLoS genetics 9(9) (2013)

Alexa Fluor® 488 goat anti-human IgG (goat, FC/IF/ICC/IHC/IHC(F)/MISC, A11013, -, -, Thermo Scientific Pierce Antibodies)
Species reactivity: Human
Application: FC/IF/ICC/IHC/IHC(F)/MISC
Citation: Waizenegger IC et al., Two distinct pathways remove mammalian cohesin from chromosome arms in prophase and from centromeres in anaphase. Cell 103(3):399-410 (2000)

Eukaryotic cell lines

Policy information about cell lines

Cell line source(s)	HeLa and MCF7 cells were purchased from ATCC.
Authentication	The Fingerprinting of all cell lines by 'AmplFLSTR identifiler PCR Amplification kit' was tested on July 12, 2019 in Korean Cell Line Bank.
Mycoplasma contamination	All cell lines tested negative for mycoplasma contamination.
Commonly misidentified lines (See ICLAC register)	No commonly misidentified cell lines were used.

Clinical data

Policy information about clinical studies

All manuscripts should comply with the ICMJE guidelines for publication of clinical research and a completed CONSORT checklist must be included with all submissions.

Clinical trial registration	The study did not involve clinical trial.
Study protocol	The study did not involve clinical trial.
Data collection	The study did not involve clinical trial.
Outcomes	The study did not involve clinical trial.

Flow Cytometry

Plots

Confirm that:

- [x] The axis labels state the marker and fluorochrome used (e.g. CD4-FITC).
- [x] The axis scales are clearly visible. Include numbers along axes only for bottom left plot of group (a 'group' is an analysis of identical markers).
- [x] All plots are contour plots with outliers or pseudocolor plots.
- [x] A numerical value for number of cells or percentage (with statistics) is provided.

Methodology

Sample preparation	Cells were fixed with 70% ethanol, permeabilized with 0.25% Triton X-100, and stained with propidium iodide.
Instrument	BD FACS Calibur 220 AVR
Software	FlowJo
Cell population abundance	After staining DNA with PI, FlowJo software was used.
Gating strategy	After staining DNA with PI, FlowJo software was used. For mitotic index, shifted phospho-MPM2 positive cells were gated.

- [ ] Tick this box to confirm that a figure exemplifying the gating strategy is provided in the Supplementary Information.
