# Supplementary material for: PRMT6-mediated H3R2me2a guides Aurora B to chromosome arms for proper chromosome segregation
Source: Nat Commun. 2020 Jan 30;11:612. doi: 10.1038/s41467-020-14511-w (PMC6992762; doi:10.1038/s41467-020-14511-w)

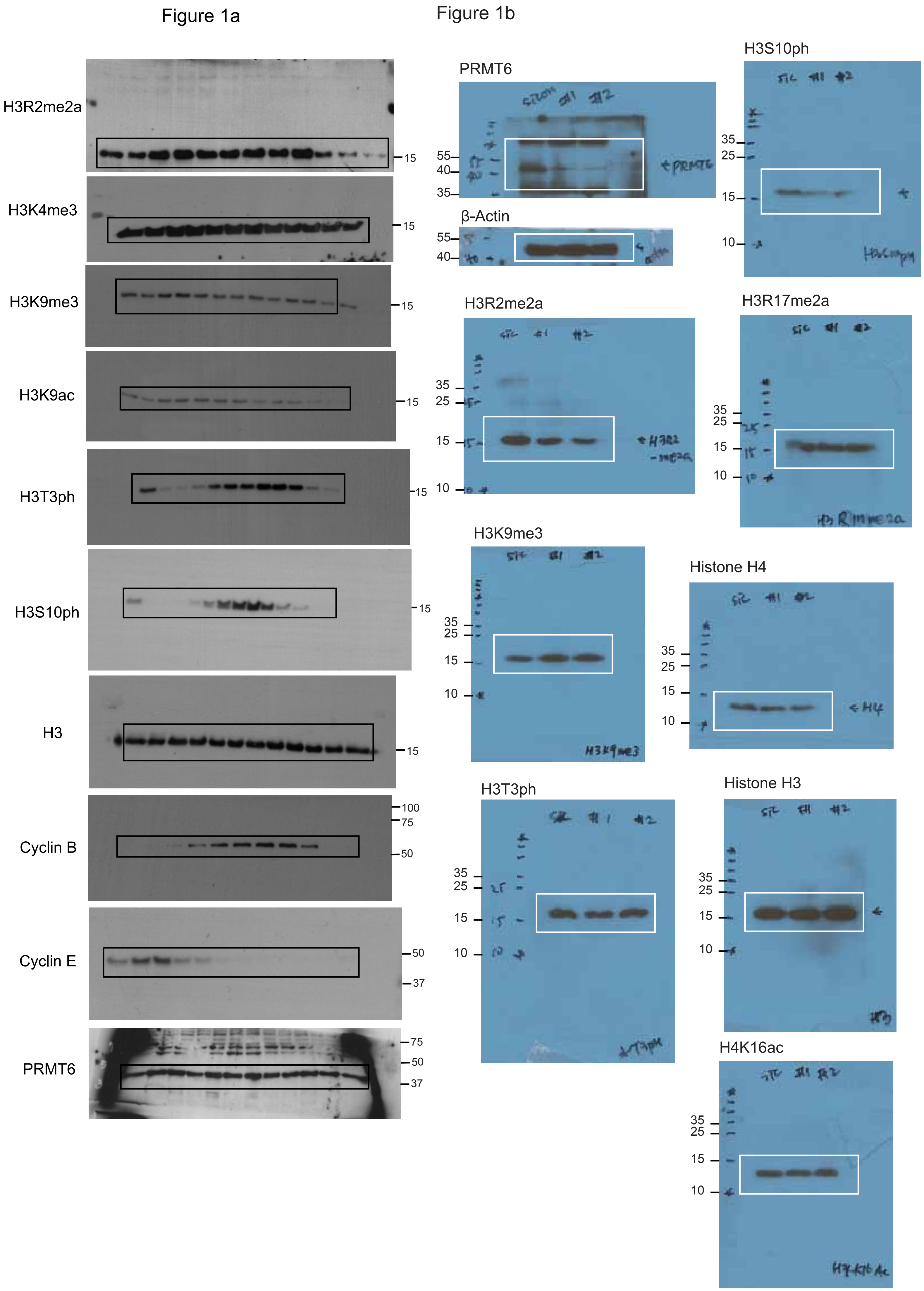

Figure 1a
Figure 1b
H3R2me2a
H3K4me3
H3K9me3
H3K9ac
H3T3ph
H3S10ph
H3
Cyclin B
Cyclin E
PRMT6
β-Actin
H3R17me2a
Histone H4
Histone H3
H4K16ac

Figure 4e

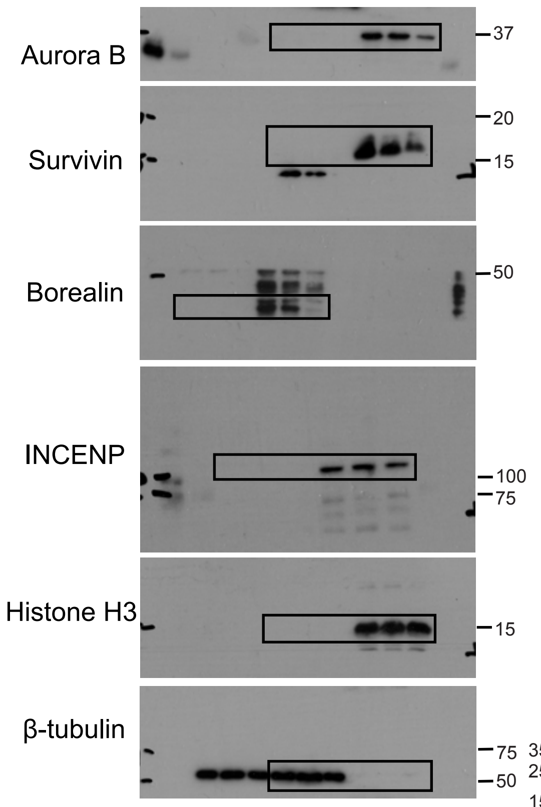

Figure 5b

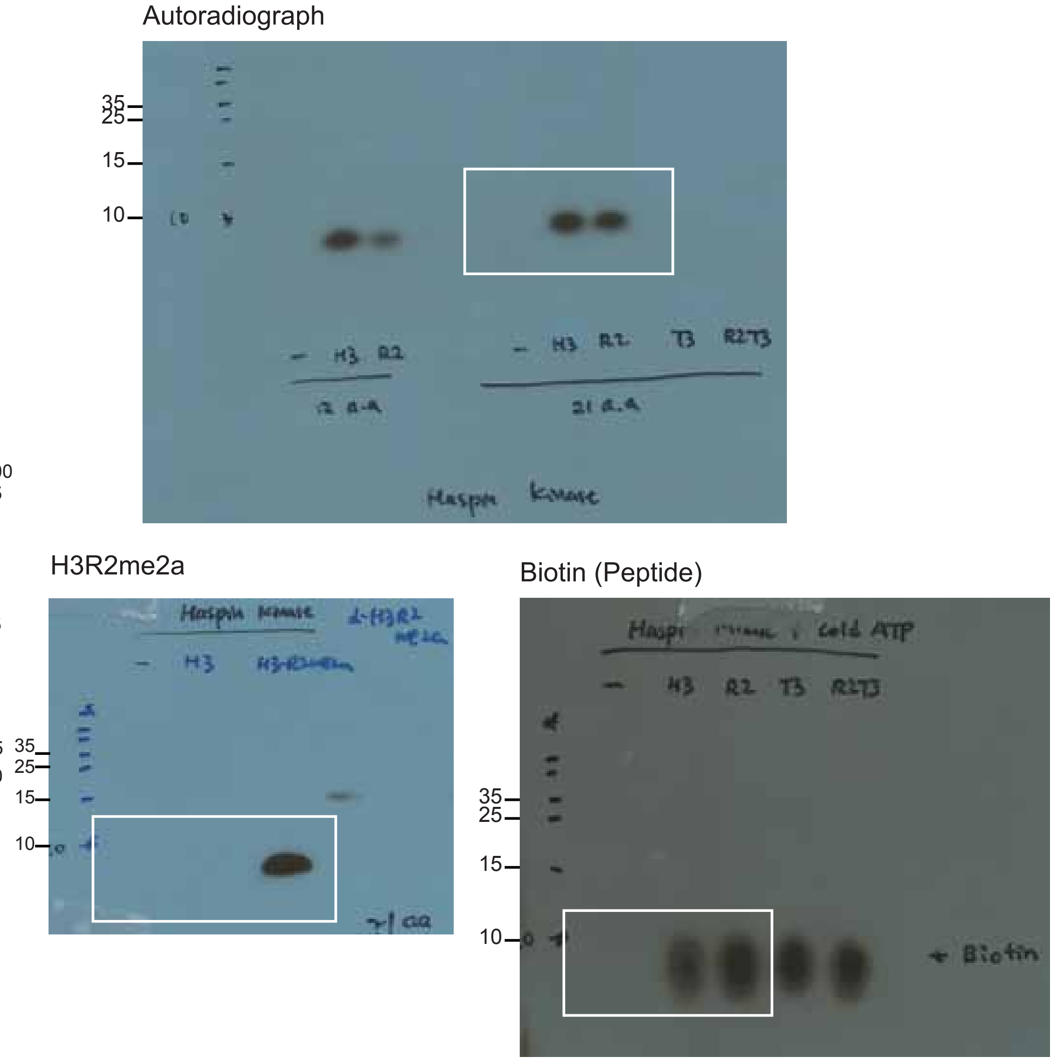

Figure 5c

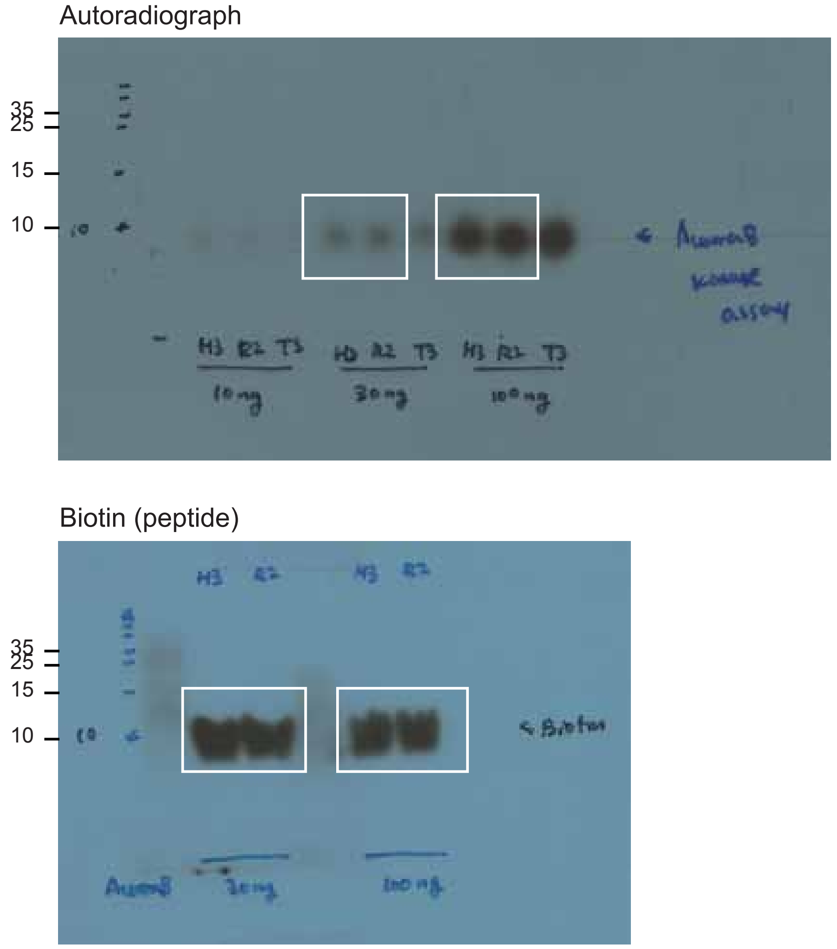

Figure 5d

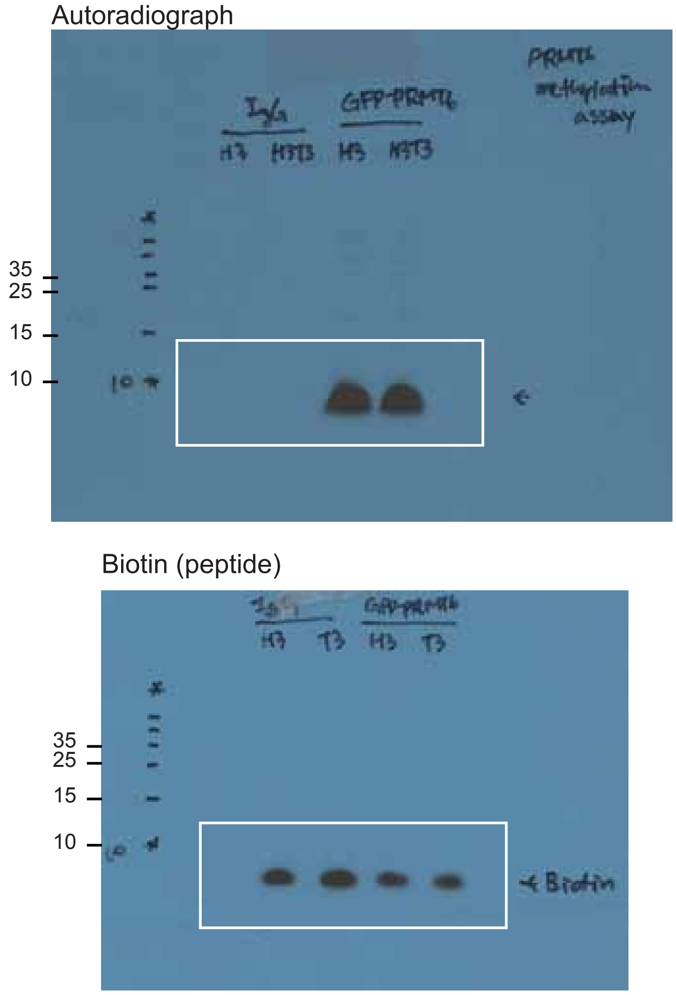

Figure 5j

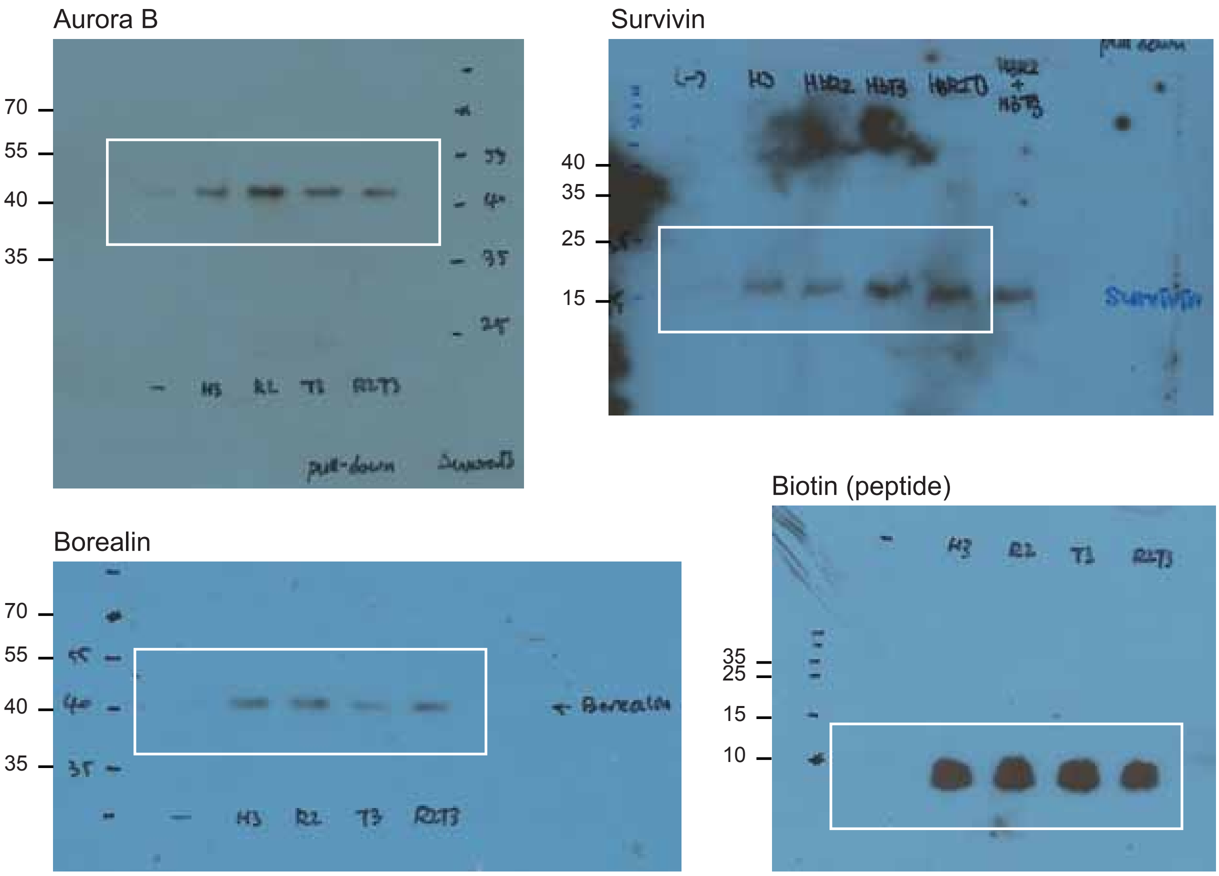
Aurora B
70
55
40
35
Survivin
40
35
25
15
Borealin
70
55
40
35
Biotin (peptide)
35
25
15
10

Figure 5k

INCENP

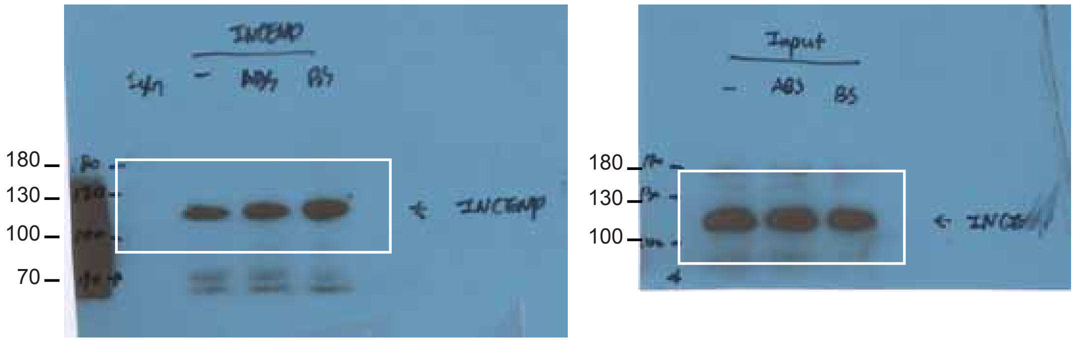

Aurora B

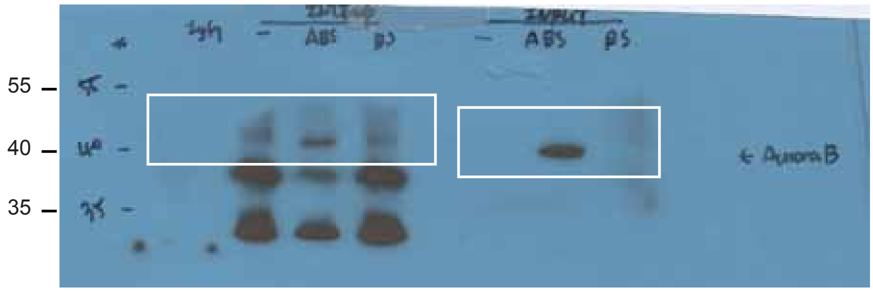

Borealin

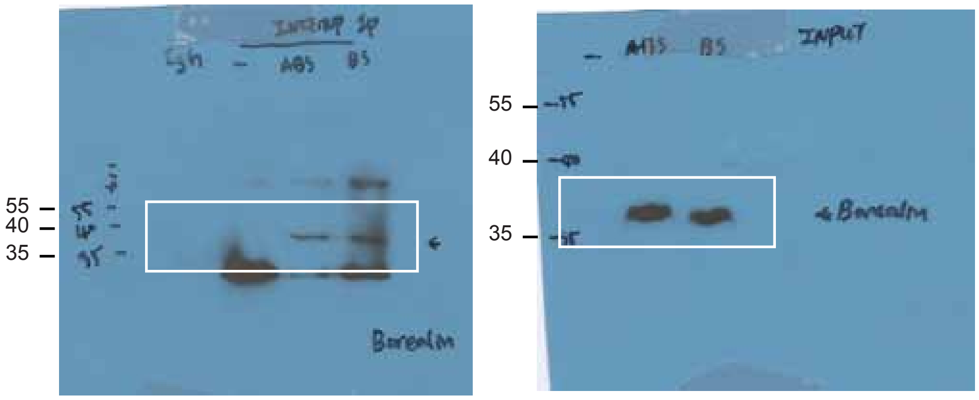

Survivin

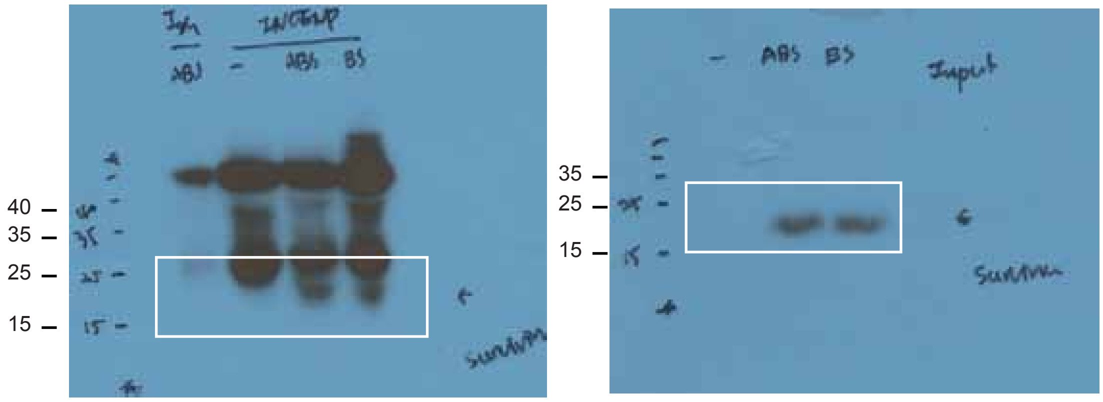

Peptide

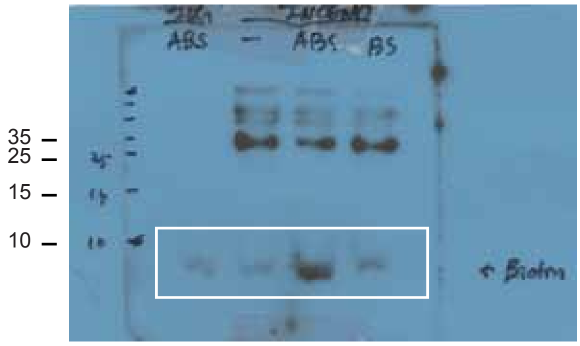

Figure 5I

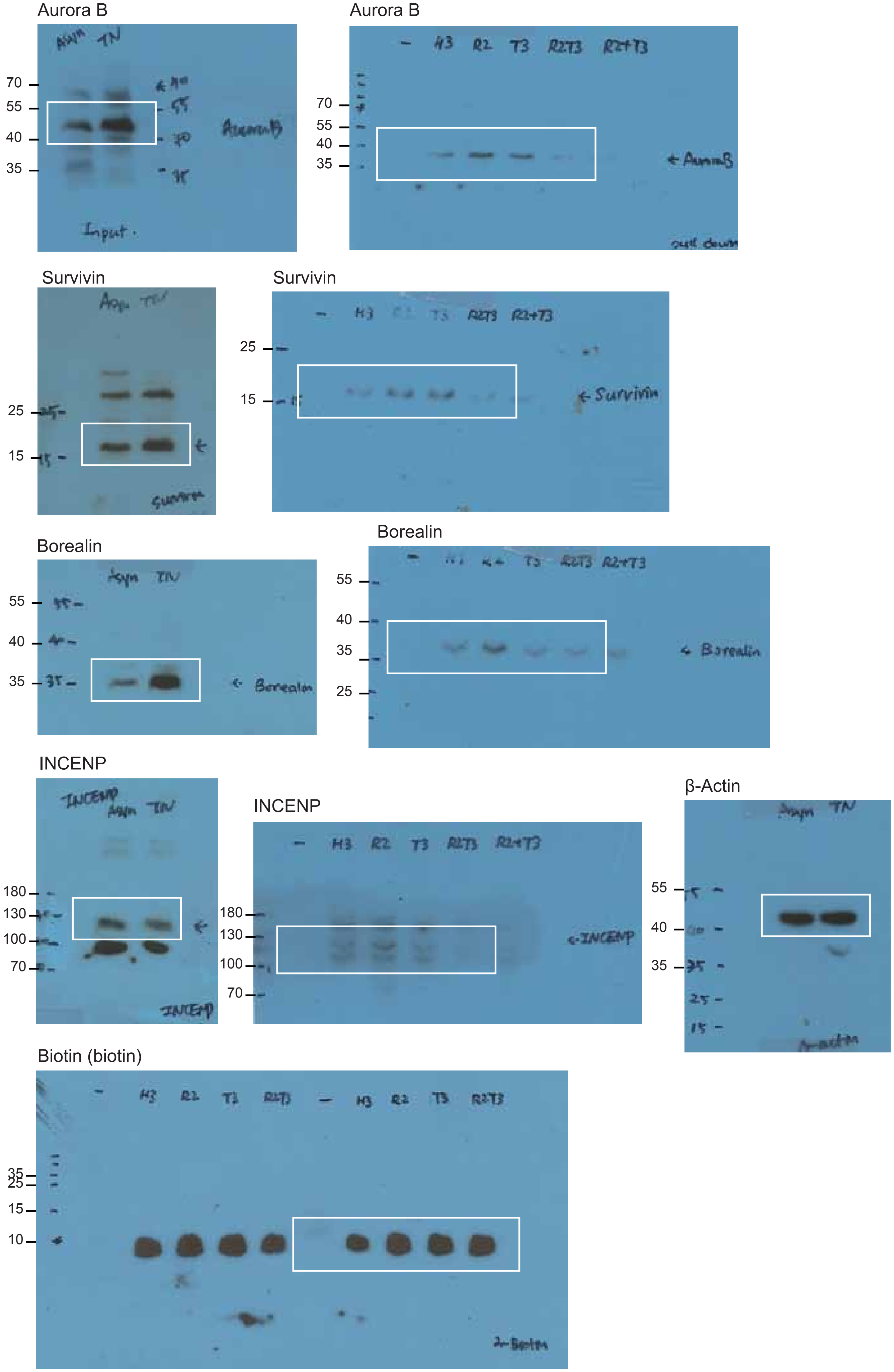
Aurora B
Aurora B
Survivin
Survivin
Borealin
Borealin
INCENP
INCENP
β-Actin
Biotin (biotin)

Supplementary Figure 1b

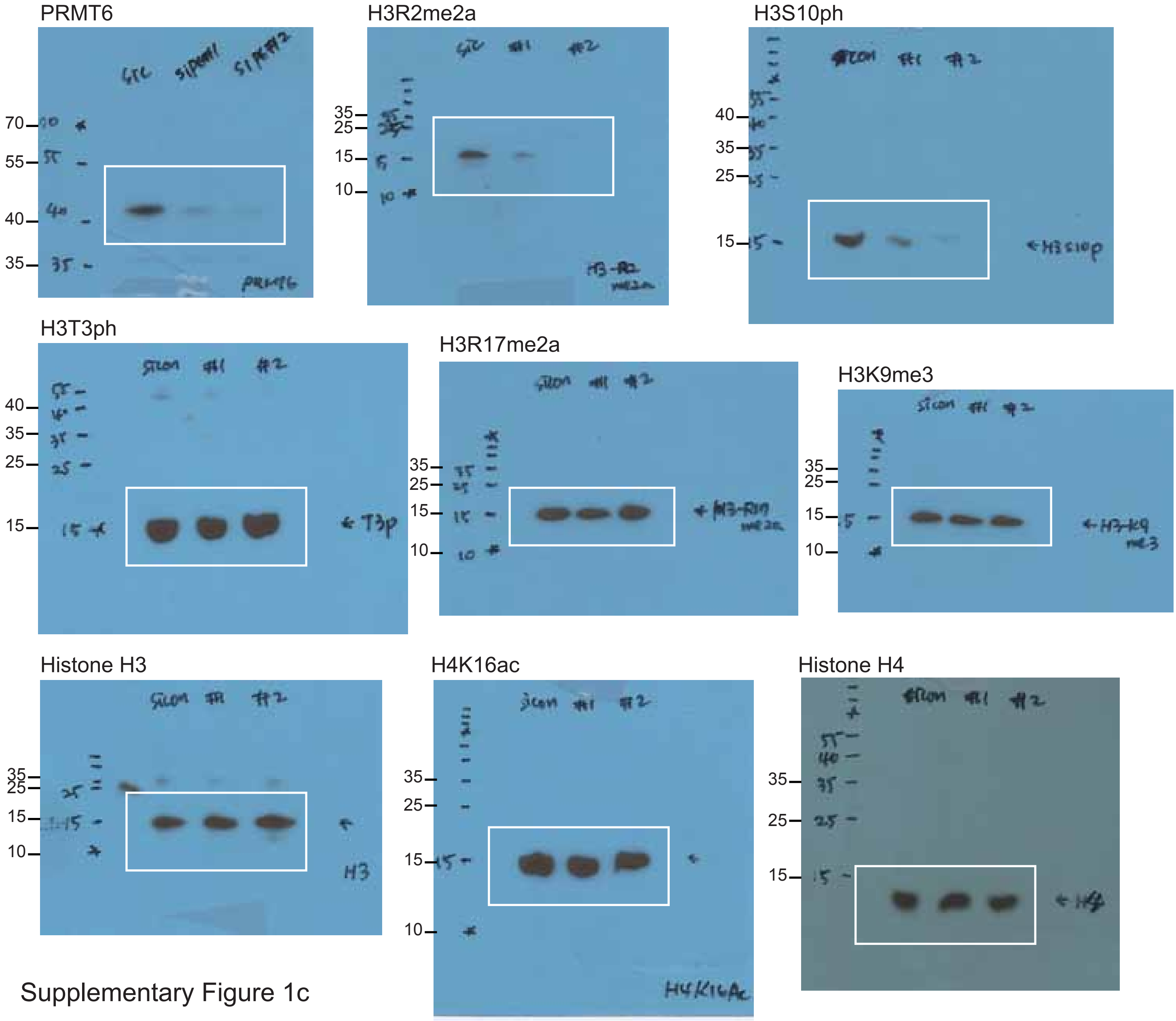

Supplementary Figure 1c

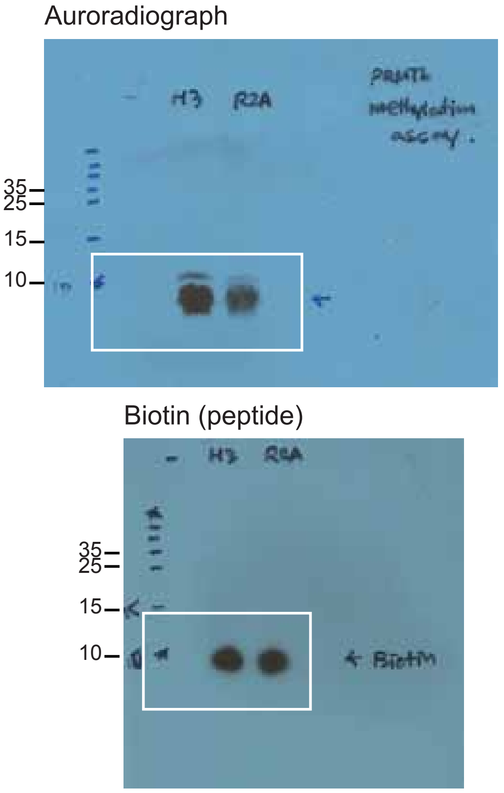

Supplementary Figure 2b

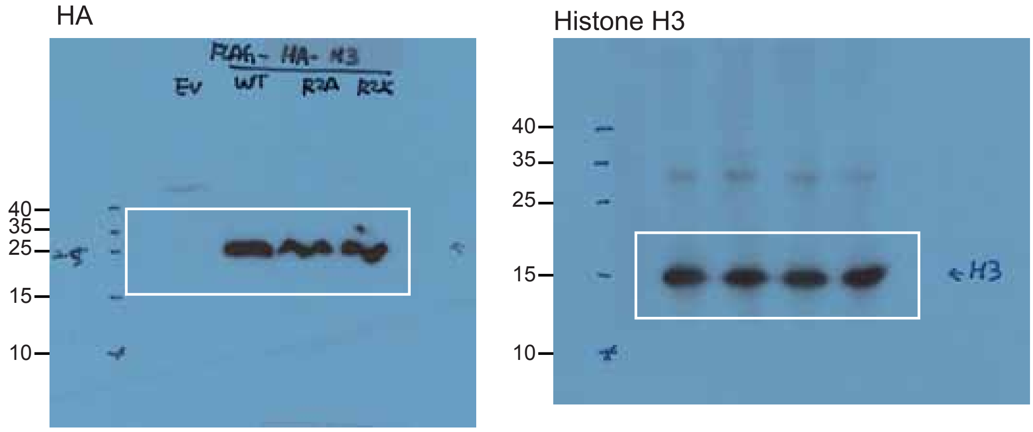

Supplementary Figure 3a

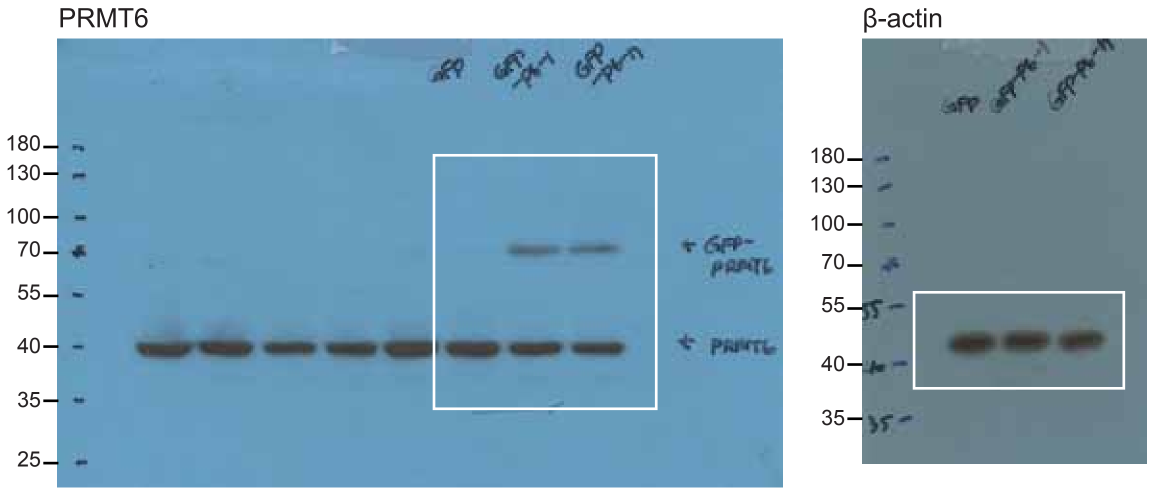

Supplementary Figure 3c

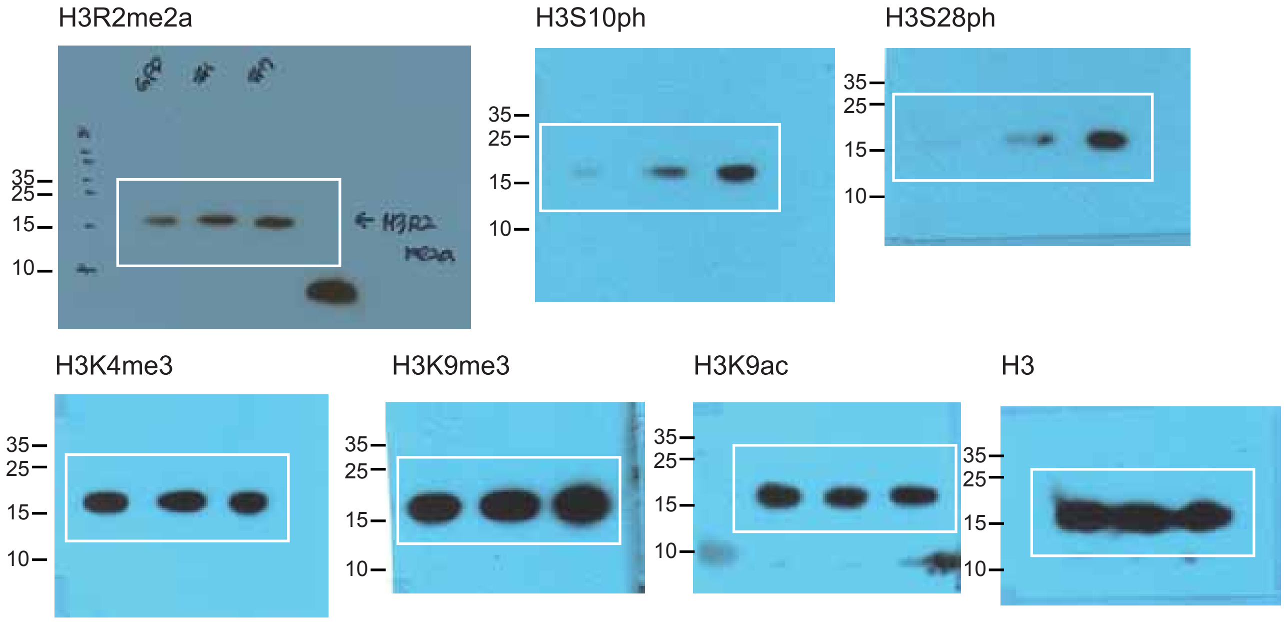

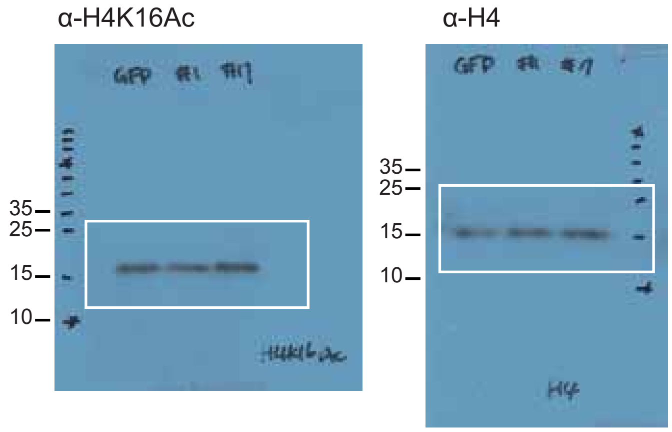

Supplementary Figure 3f

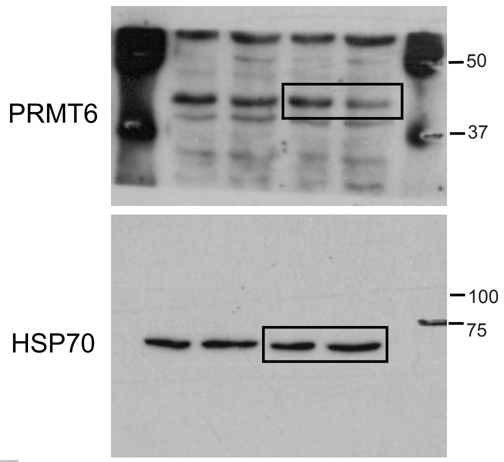

Supplementary Figure 4c

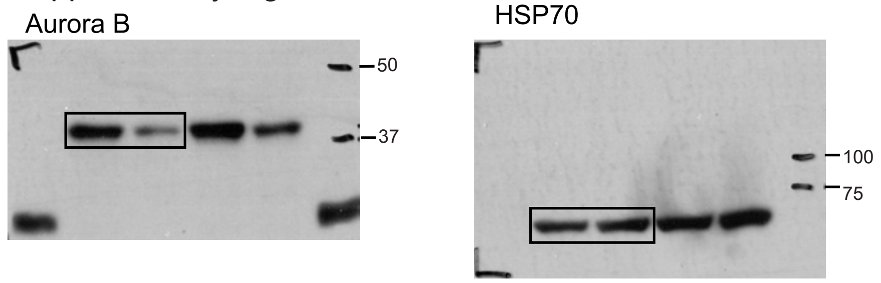

Supplementary Figure 6d

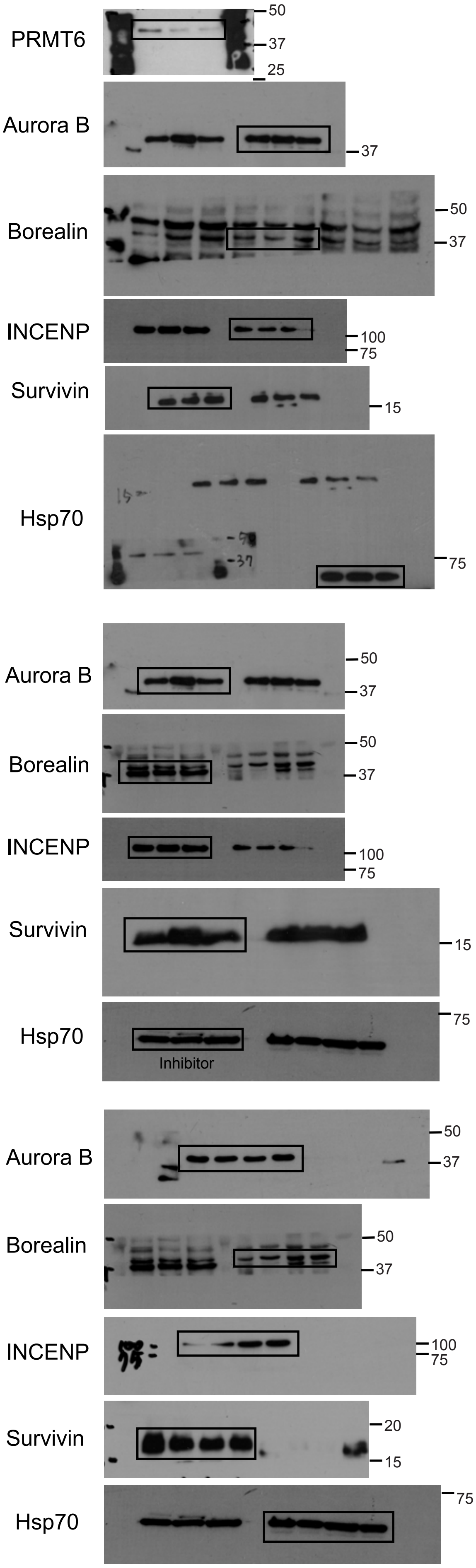

Supplementary Figure 8a

Autoradiograph

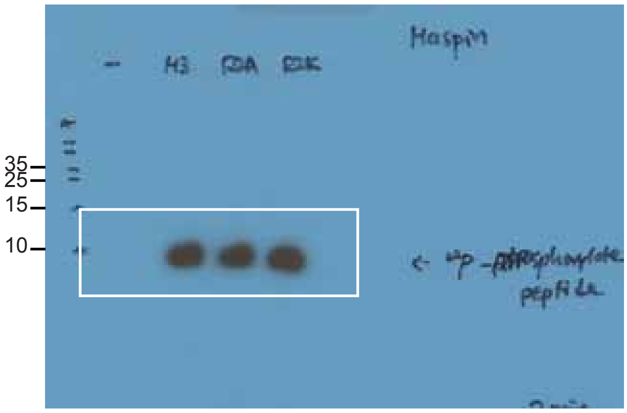

H3T3ph

Biotin (peptide)

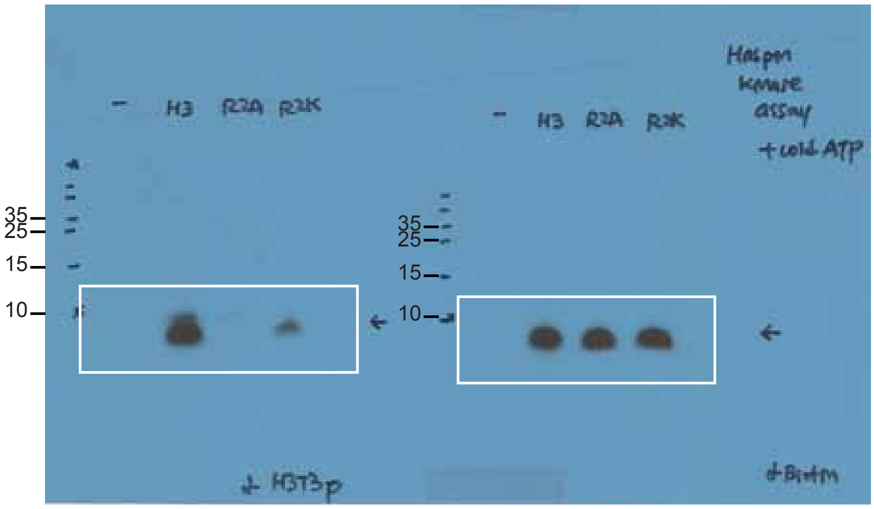

Supplementary Figure 10a

Autoradiograph

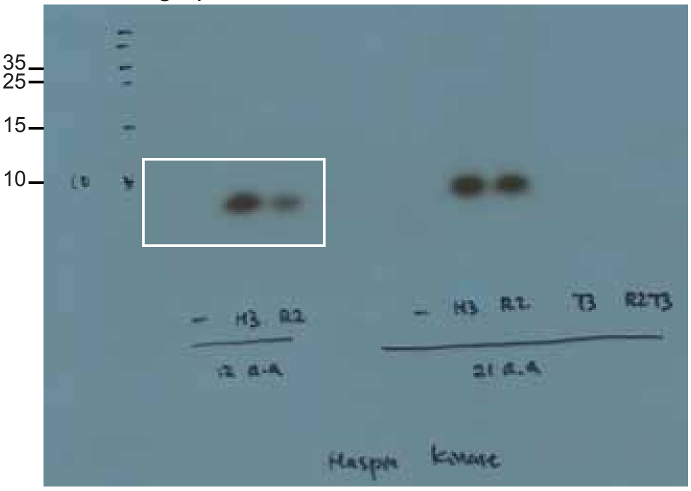

H3R2me2a

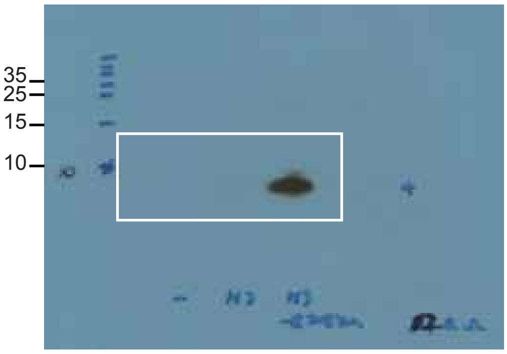

Ponceau S staining (peptide)

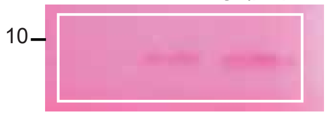

Supplementary Figure 10b

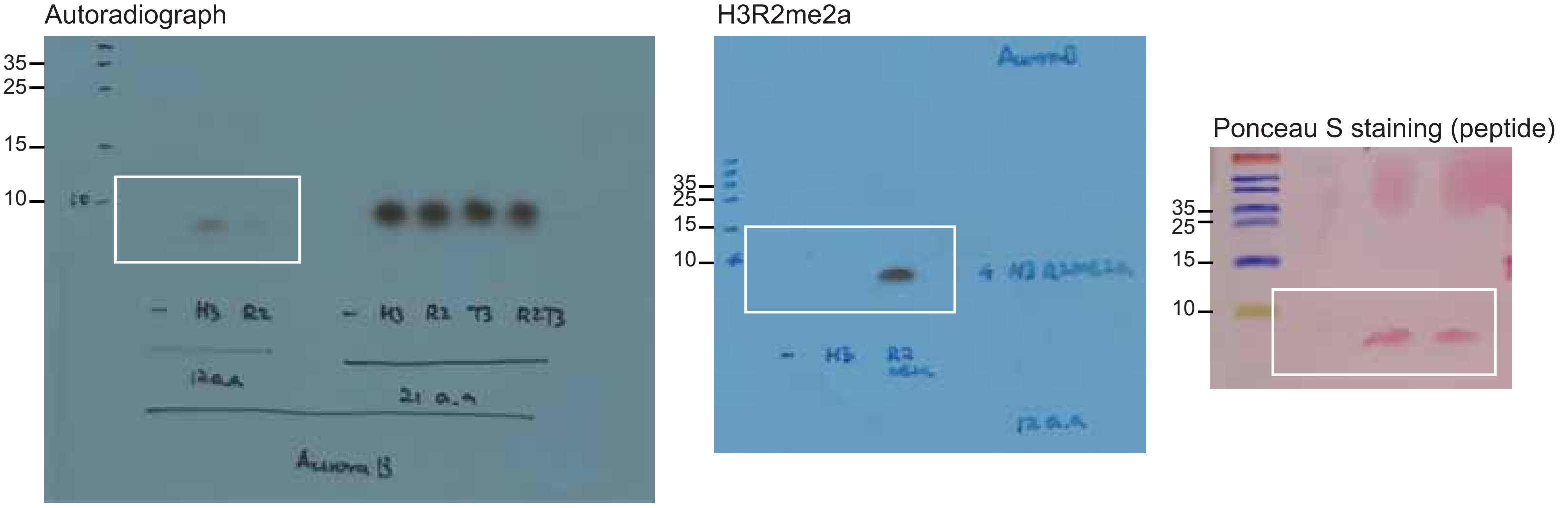

Supplementary Figure 10c

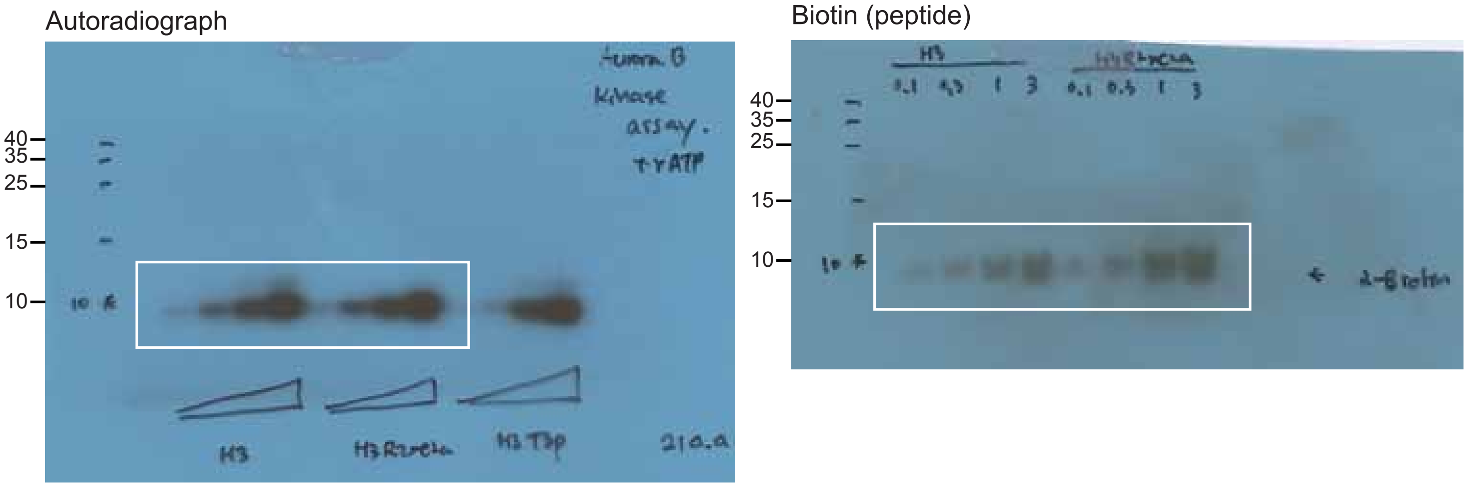

Supplementary Figure 10d

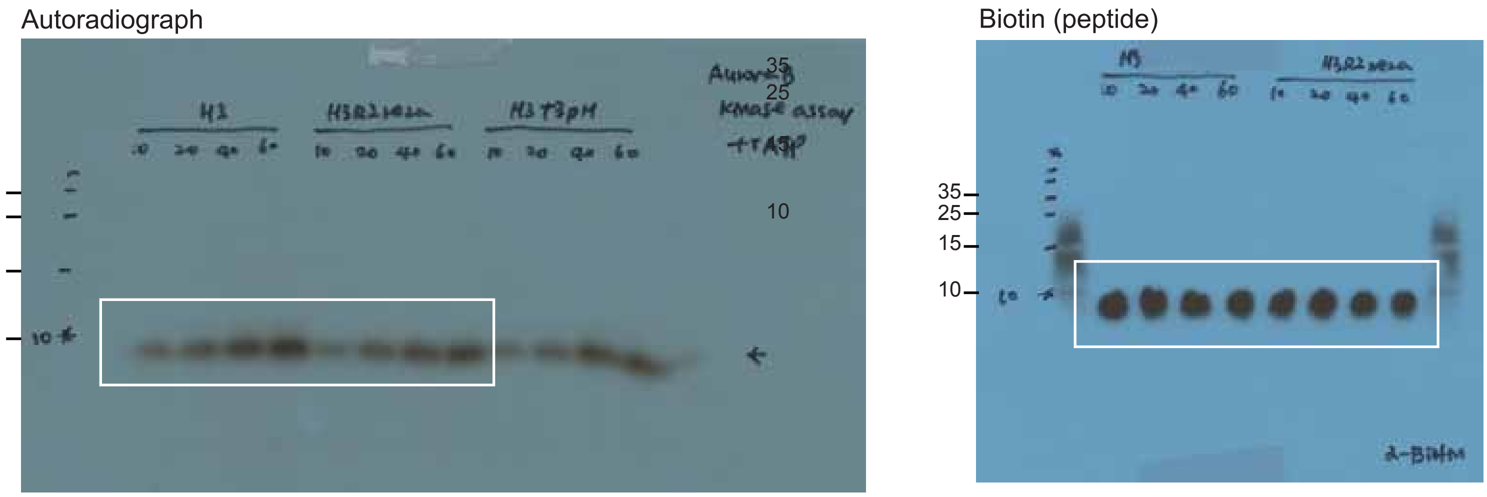

Supplement: Supplementary file 15 — Source Data [file 41467_2020_14511_MOESM15_ESM.zip › Source Data - Uncropped Blots.pdf]
